# Non-Destructive Hardness Indentation Measurement of Residual Stress on Large Aerospace Forged Components at the Engineering Site Based on Impact Hardness Tester

**DOI:** 10.3390/ma17143436

**Published:** 2024-07-11

**Authors:** Jingyuan Niu, Peiran Tian, Siao Sun, Yage Zhang, Guizeng Song, Qiang Song, Qinghua Li, Nianxuan Hu, Fuguo Li

**Affiliations:** 1 State Key Laboratory of Solidification Processing, School of Materials Science and Engineering, Northwestern Polytechnical University, Xi’an 710072, China; 2485958832@mail.nwpu.edu.cn (J.N.); 2020261202@mail.nwpu.edu.cn (Y.Z.); jinyuan@mail.nwpu.edu.cn (G.S.); sq103109@mail.nwpu.edu.cn (Q.S.); qinghual@nwpu.edu.cn (Q.L.); 2021261125@mail.nwpu.edu.cn (N.H.); 2 Queen Mary University of London Engineering School, Northwestern Polytechnical University, Xi’an 710072, China; 2021304252@mail.nwpu.edu.cn (P.T.); siaosun@yeah.net (S.S.); 3 Shaanxi Key Laboratory of High-Performance Precision Forming Technology and Equipment, Northwestern Polytechnical University, Xi’an 710072, China

**Keywords:** forgings, residual stress, impact indentation, dimensionless function, reverse algorithms

## Abstract

Large forgings are crucial in aerospace applications; however, the residual stresses generated during their forming and heat treatment seriously affect their serviceability. Therefore, the non-destructive detection of residual stresses in large forgings is of far-reaching significance for ensuring the quality of forgings and realising precision machining. Although a variety of detection methods are available, there is still a lack of a programme that can comprehensively, accurately and non-destructively measure the residual stresses in large forgings. This study is dedicated to exploring the application of the bouncing impact indentation method in the non-destructive testing of residual stresses in large forgings. Through in-depth finite element simulations and orthogonal scheme analyses, we found that the elastic modulus, yield strength and work hardening indexes have significant effects on the impact indentation process. Further, we establish the dimensionless function of residual stress and indentation parameters, and successfully obtain the inversion algorithm of residual stress. The relative error of the calculated values of the indentation curves hm and hr in the simulation with reference values is not more than 3%, and the relative error of the corrected Pm inversion values for most virtual materials is not more than 5%. The folding elastic modulus and apparent elastic modulus obtained by inversion are controlled within 10%, which demonstrates a high value for engineering applications. In addition, we innovatively express the research results in the form of 3D stress diagrams, realising the digital expression of 3D residual stresses in large forgings based on feature point measurements and contour surface configurations, which provides intuitive and comprehensive data support for engineering practice.

## 1. Introduction

Residual stress is defined as the stress that still exists inside the material and reaches self-equilibrium under the action of no applied load [1]. Macroscopic residual stresses in the material are mainly due to the following three reasons [2]: uneven plastic deformation, thermal effects and chemical effects; microscopic residual stresses in the material exist for the following three reasons: due to the anisotropy of the modulus of elasticity and the coefficient of thermal expansion of different grains, due to an intra-granular and inter-granular slip or the formation of twins, due to the generation of different phases within the material, resulting in changes in the volume, and so on [2].

The integration of large forgings in the field of civil and military aviation has a wide range of applications [1]; however, the residual stress introduced in the manufacturing process will seriously reduce the dimensional stability, fatigue resistance and corrosion resistance of the forging, etc, and with the integration of large forgings, as well as the precision manufacturing and large-scale application of numerical control integrated machining methods, the impact of residual stress will become more and more obvious. Therefore, the measurement and characterization of residual stresses within the material becomes particularly important.

In engineering practice, the characterization of residual stresses is broadly divided into two methods: mechanical release detection and non-destructive testing [3]. The mechanical release testing method, also known as the stress release method, refers to the use of mechanical means of the material under test from the mechanical structure of a part of the split, and the study of the degree of deformation of the local area to determine the size of the residual stress. The mechanical release test method is also divided into blind hole method, layer by layer milling method, ring cutting method, contour method, etc., [4,5]. These methods have mature theory, high measurement accuracy and high measurement efficiency; however, these methods need to destroy the specimen, for some of the fine near forming as well as some of the parts in service cannot be detected. Non-destructive testing, also known as physical testing, is a method of characterizing residual stresses in materials by detecting changes in the physical and chemical properties of the material. Non-destructive testing methods include X-ray diffraction, ultrasonic methods, and neutron diffraction [4,5]. These physical testing methods are often more accurate than mechanical release methods and do not damage the workpiece; however, their drawbacks are the harsh testing conditions and the complexity and high cost of the testing process. In addition, the non-destructive testing method is usually sensitive to grain size and microstructure, and requires the preparation of stress-free reference specimens for comparative testing—generally, the laboratory of scientific research institutes have the appropriate testing conditions—and production site testing is more difficult.

As a simple, fast and minimally destructive testing method, the indentation method has been widely used to characterize the mechanical behavior of materials in recent years [6]. The indentation test refers to taking a certain shape of the indenter pressed into the surface of the material, and when the indenter unloading can obtain a certain depth of the indentation and indentation area, the load–displacement curve can be derived from some of the basic mechanical properties of the material parameters through a series of calculations, such as Young’s modulus, yield strength, hardness, and work hardening index and so on [6].

At present, domestic and foreign research, and the results for the indentation method, are as follows: Frankel [7] found that the residual stress is approximately inversely proportional to the Rockwell hardness, and Underwood [8] found that the phenomenon of material buildup around the indentation can characterize the residual stress, and optical interferometric measurements of the buildup shape can be used to calculate the residual stress. Since 1992, Oliver and Pharr [9,10,11] have developed the theory of indentation testing and proposed a data processing method for determining the modulus of elasticity and hardness of a material using unloaded curvature, which has promoted the rapid development of indentation testing methods. To avoid the use of reference samples, Cheng [12] proposed a new method combining gauge analysis and finite element simulation. Hongping Jin [13] carried out gauge analysis and finite element simulation for spherical indentation and proposed a method to measure indentation hardness and residual stress based on the energy principle. Yuan Lingxiao [14] found that the residual stress has a significant effect on the material hardness. Indentation methods are classified as static load and impact type, and impact indentation has also been proved to be feasible. Mijailovic, Aleksandar S. et al. [15] proposed an analytical model of impact indentation for the measurement of the visco–elastic modulus and relaxation time constant of highly pliable polymers and bio-soft tissues. Lei Shen [16] investigated the relationship between indentation bulge and residual stress by experiment and simulation, and proposed a method of residual stress characterization by measuring the bulge around the indentation instead of the indentation depth. Therefore, the impact indentation method can draw on the theoretical study of the conventional indentation method and combine it with simulation to obtain the residual stress magnitude.

Recently, the indentation instrument has been rapidly developed in commercial applications, although the use of the indentation method to characterize residual stress from the theoretical model, as well as finite element simulation used in experimental research, has limitations in all aspects of development. However, the theory of this model is still not perfect or applicable to actual scenarios due to the lack of a unified standard for the parameter settings of the dynamic simulation of impact indentation. The use of the impact method for characterizing and testing residual stress on the surface of the specimen is not perfect and has limitations. Combined with the Leeb hardness tester impact indentation method used in this paper, there is also the energy distribution of impact indentation that needs to be quantitatively calculated, the force–energy–displacement curve of impact indentation needs to be quantitatively expressed, and the relationship between the changes of elastic modulus and other characteristic parameters, and the evolution of the residual stress, needs to be quantitatively described [17].

Based on the study, analysis and summary of existing indentation theories, this paper, combined with the special needs of aerospace forgings, carries out research on the residual stress testing of impact indentation, as well as the characterization and engineering practices; based on the Leeb hardness tester [17], we explore the inverse calculation method of the basic characteristic parameters of the material under the action of the impact loading, and the characterization calculation of the residual stresses under the universal material model. Using finite element simulation and other comparable methods to measure the residual stress of the workpiece, the residual stress measured by the impact indentation method is compared and corroborated to improve the accuracy of the residual stress characterization of the dynamic impact indentation method, and the feasibility of its implementation is explored in conjunction with the engineering applications, so as to provide the basis for the measurement of the residual stress by the impact indentation method and the application of the preliminary exploration of the residual stress of the forging characteristic points, from the surface to the inside of the structure. It is also used to explore the three-dimensional residual stress distribution of forgings based on the residual stress at the characteristic points of forgings, so as to provide a reference for the study of the formation and distribution law of residual stress in forgings.

## 2. Methods

### 2.1. Theoretical Model Analysis

#### 2.1.1. Indentation Analysis Curve

**Figure 1:**
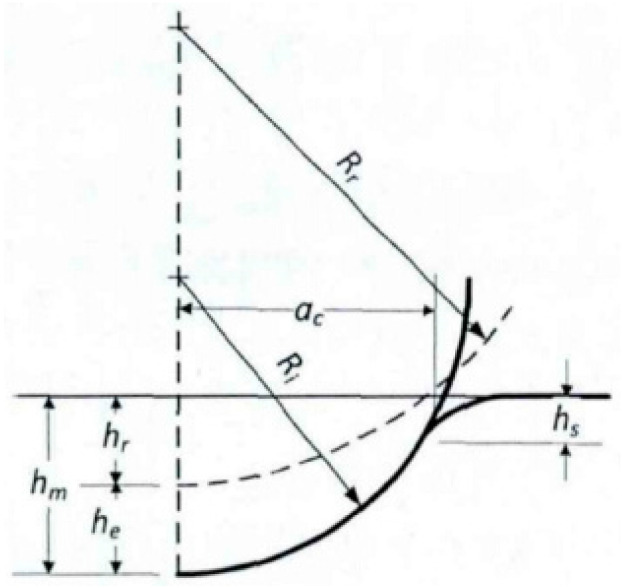
Indentation of sphere and half space deformable body.

The classical solution of Hertz’s theory forms the basis of much experimental and theoretical work in the field of contact mechanics, and provides a framework for analyzing the effects of non-rigid indenters [18]. In studying the contact problem of an elastic sphere with an elastic half-space, it is assumed that both contacting bodies are assumed to be elastic half-spaces, the contact stresses above and below the contact region are assumed to be equal, and the contact region is small compared to the spherical indenter, resulting in the projection of the contact region as a circle. For example, Figure 1 shows a schematic diagram of the indentation of a sphere by a deformed body in half-space.

Assume that the pressure in the contact area is distributed in a parabola:

(1) $$p=p_{0}\sqrt{1-{\left(r/a_{c}\right)}^{2}}$$

where *p*_0_ is the maximum contact stress of the contact center; *r* is the radial distance between the contact point and the contact center; *a*_c_ is the circular radius of the contact area.

By integrating the above formula within the radius 0–*a*_c_ of the entire contact area, the resultant force generated by the two contacts can be obtained:

(2) $$F=\frac{2}{3}p_{0}\pi a_{c}^{2}$$

The expression of the contact radius *a*_c_ is:

(3) $$a_{c}={\left(\frac{3FR}{4E^{*}}\right)}^{1/3}$$

where E* is the reduced elastic modulus, which is determined by the material properties of the two contacts:

(4) $$\frac{1}{E^{*}}=\frac{1-v^{2}}{E}+\frac{1-v_{i}^{2}}{E_{i}}$$

where *v* and *E* are the Poisson’s ratio and elastic modulus of the measured material, respectively; *v*_i_ and *E*_i_ are the Poisson’s ratio and elastic modulus of the ball-shaped indenter, respectively.

Sneddon [19] deduced the general relationship between the displacement, load and contact area of any indenter which can be described as a smooth functional rotation body, and showed that the load–displacement relationship of many simple indenter geometers can be expressed by the following formula:

(5) $$P=\alpha h^{m}$$

where *P* is the indentation load; *h* is the displacement; *α*, *m* are parameters related to materials constants.

**Figure 2:**
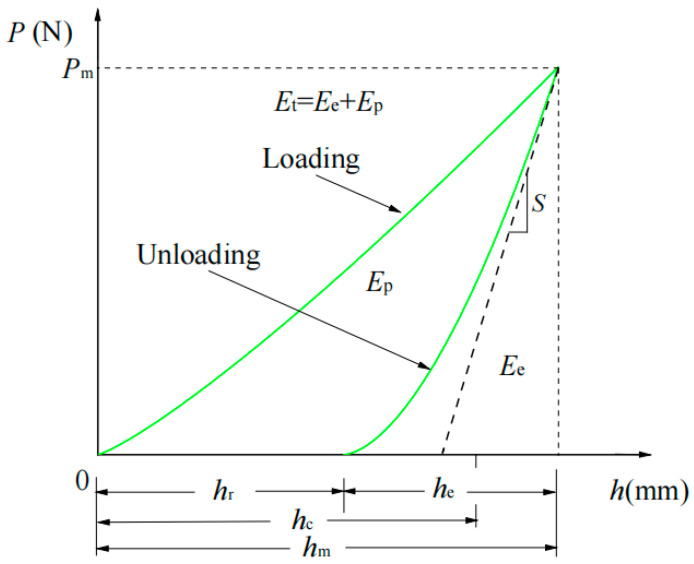
Schematic diagram of indentation load–displacement curve.

In the actual indentation test loading process, elastic deformation occurs first, then plastic deformation occurs; the unloading stage is considered to be a complete elastic deformation behavior, and the core indentation shape after the spherical indenter is completely unloaded is considered to be spherical. Generally, the load displacement curve of the indentation process is shown in Figure 2, where *h*_m_ is the indentation depth; *h*_r_ is the depth of residual indentation; *R*_i_ is defined as the radius of the sphere; *R*_r_ is the radius of spherical crater of residual indentation; and *h*_s_ is the height of subsidence or uplift.

(6) $$h_{c}=\frac{h_{m}+h_{r}}{2}$$

(7) $$h_{m}=h_{s}+h_{c}$$

(8) $$a_{c}=\sqrt{R_{i}^{2}-h_{c}^{2}}$$

where *h*_c_ is the contact depth.

By analyzing the *P*–*h* curve, the expression of loading curve and unloading curve can be obtained as follows:

(9) $$P=kh^{\alpha }$$

(10) $$P=k^{\prime }{\left(h-h_{r}\right)}^{\beta }$$

The corresponding energy expression can be obtained by integrating the loading curve and unloading curve accordingly:

(11) $$E_{e}=\frac{P_{m}(h_{m}-h_{r})}{\beta +1}$$

(12) $$E_{t}=\frac{P_{m}h_{m}}{\alpha +1}$$

where *E*_e_ is the elastic energy; *E*_p_ is the plastic energy; *E*_t_ is the total energy, *E*_p_ = *E*_t_ − *E*_e_.

#### 2.1.2. Virtual Material Model

The material constitutive model is the inherent property of the material, and it is the basis for mechanical analysis in the simulation and testing process.

(13) $$\sigma =\left\{\begin{array}{cc} E\varepsilon , & \sigma \leq \sigma_{y}\\ K\varepsilon^{n}, & \sigma \geq \sigma_{y} \end{array}\right.$$

where *E* is the elastic modulus; *σ*_y_ is the initial yield strength; *n* is the work hardening index.

According to the continuity condition, it is known that the strength coefficient *K* = *σ*_y_ (*E*/*σ*_y_)^n^, which is substituted into the formula, can be obtained as follows:

(14) $$\sigma =\left\{\begin{array}{cc} E\varepsilon , & \sigma \leq \sigma_{y}\\ \sigma_{y}{\left(1+\frac{E}{\sigma_{y}}\varepsilon_{p}\right)}^{n}, & \sigma \geq \sigma_{y} \end{array}\right.$$

where *ε*_p_ is the plastic strain.

#### 2.1.3. Tensor Representation Model

When the stress state of any point is fully described in terms of a tensor, the specific form of the stress tensor can be expressed by a symmetric matrix:

(15) $$\sigma_{\mathrm{ij}}=\left(\begin{array}{ccc} \sigma_{x} & \tau_{\mathrm{xy}} & \tau_{\mathrm{xz}}\\ \tau_{\mathrm{yx}} & \sigma_{y} & \tau_{\mathrm{yz}}\\ \tau_{\mathrm{zx}} & \tau_{\mathrm{zy}} & \sigma_{z} \end{array}\right)$$

(16) $$\left(\begin{array}{ccc} \sigma_{x} & \tau_{\mathrm{xy}} & \tau_{\mathrm{xz}}\\ \tau_{\mathrm{yx}} & \sigma_{y} & \tau_{\mathrm{yz}}\\ \tau_{\mathrm{zx}} & \tau_{\mathrm{zy}} & \sigma_{z} \end{array}\right)=\left(\begin{array}{ccc} \sigma_{x}-\sigma_{m} & \tau_{\mathrm{xy}} & \tau_{\mathrm{xz}}\\ \tau_{\mathrm{yx}} & \sigma_{y}-\sigma_{m} & \tau_{\mathrm{yz}}\\ \tau_{\mathrm{zx}} & \tau_{\mathrm{zy}} & \sigma_{z}-\sigma_{m} \end{array}\right)+\left(\begin{array}{ccc} \sigma_{m} & 0 & 0\\ 0 & \sigma_{m} & 0\\ 0 & 0 & \sigma_{m} \end{array}\right)$$

(17) $$\sigma_{m}=\frac{1}{3}(\sigma_{x}+\sigma_{y}+\sigma_{z})$$

The stress tensor at any point can be divided into two parts: the stress sphere tensor and the stress flexural tensor. The spherical stress tensor is positive in any direction, the stress in all directions is the same, and there is no shear stress, which can only change the elastomer of the material.

The geometric expression form will be more intuitive and clearer; for example, the stress Mohr circle can directly obtain the magnitude and symbol of the principal stress and the direction relationship with the actual stress, and the analysis and expression are obvious.

For the three-way stress state, assuming that the relationship between the three principal stresses is *σ*_1_ ≥ *σ*_2_ ≥ *σ*_3_, then the stress at one point should satisfy the formula:

(18) $${\left(\sigma_{m}-\frac{\sigma_{1}+\sigma_{3}}{2}\right)}^{2}+\tau_{m}\leq {\left(\frac{\sigma_{1}-\sigma_{3}}{2}\right)}^{2}$$

(19) $${\left(\sigma_{m}-\frac{\sigma_{1}+\sigma_{2}}{2}\right)}^{2}+\tau_{m}\leq {\left(\frac{\sigma_{1}-\sigma_{2}}{2}\right)}^{2}$$

(20) $${\left(\sigma_{m}-\frac{\sigma_{2}+\sigma_{3}}{2}\right)}^{2}+\tau_{m}\leq {\left(\frac{\sigma_{2}-\sigma_{3}}{2}\right)}^{2}$$

**Figure 3:**
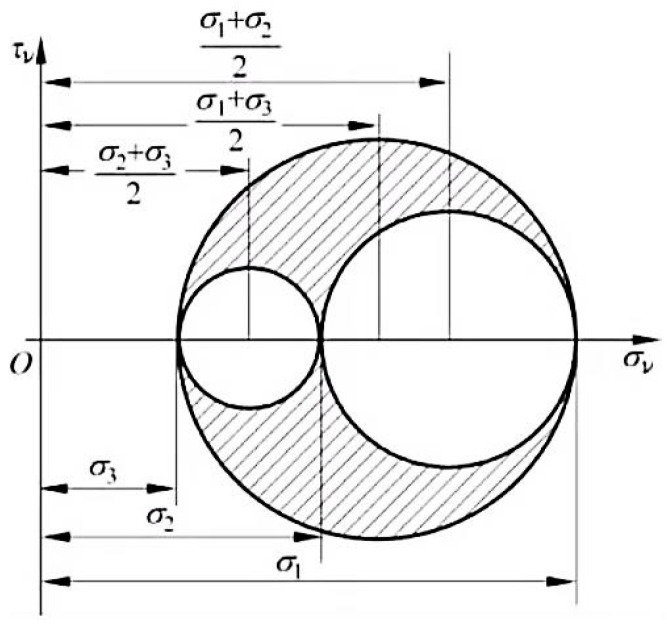
Mohr’s circle of triaxial stress.

When the equal sign of this formula is true, the three circles represented by it are called three-way stress Mohr circles, as shown in Figure 3.

According to the Mises yield condition, the equivalent stress *σ*_s_ is as follows:

(21) $${\left(\sigma_{x}-\sigma_{y}\right)}^{2}+{\left(\sigma_{y}-\sigma_{z}\right)}^{2}+{\left(\sigma_{z}-\sigma_{x}\right)}^{2}+6(\tau_{\mathrm{xy}}^{2}+\tau_{\mathrm{yz}}^{2}+\tau_{\mathrm{zx}}^{2})=2\sigma_{s}^{2}$$

or

(22) $${\left(\sigma_{1}-\sigma_{2}\right)}^{2}+{\left(\sigma_{2}-\sigma_{3}\right)}^{2}+{\left(\sigma_{3}-\sigma_{1}\right)}^{2}=2\sigma_{s}^{2}$$

**Figure 4:**
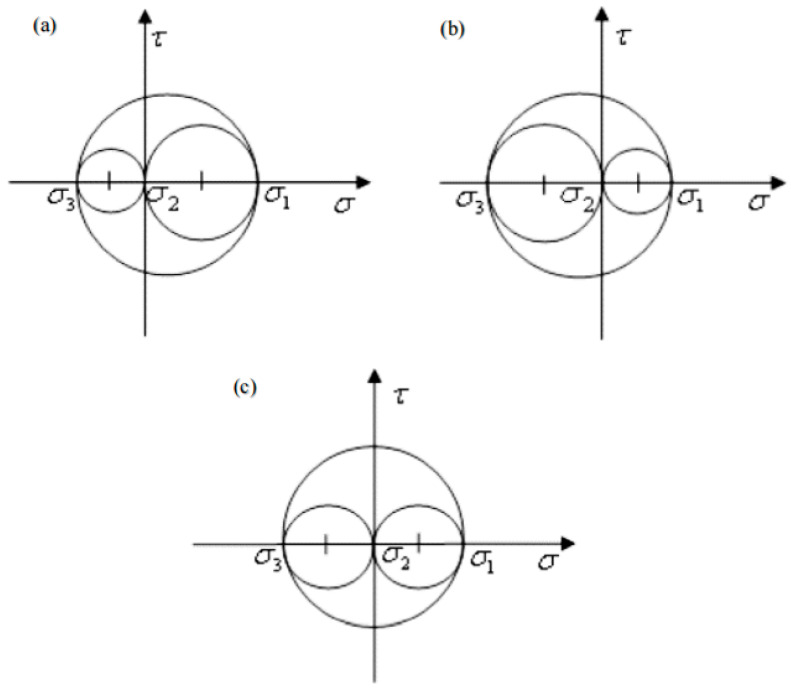
Mohr circle of triaxial stress: (**a**) generalized tension; (**b**) generalized compression; (**c**) pure shearing.

The different magnitude and direction of the three principal stresses of the deformable body will make the deformable body take different deformation stress states. The three conditions shown in Figure 4 can roughly classify the residual stress state of the forging.

### 2.2. Residual Stress Characterization

#### 2.2.1. Impact Indentation Test Method

This test is based on Leeb hardness tester (It’s made by PROCEQ, Schwerzenbach, Switzerland) to measure the elastic rebound speed of impact indentation, and calculate the apparent elastic modulus affected by residual stress according to the rebound speed [17], so as to characterize the residual stress of the forging.

The Leeb hardness tester, also known as the Leeb Rockwell hardness tester, is a commonly used instrument for measuring the hardness of metal materials [17]. The principle is the same as that of the cloth hardness tester. The difference is that it is a mechanical test method in which the tensile strength and compressive strength of the material are calculated by the measured deformation after the indenter is pressed into a certain depth on the surface of the sample. When measuring, the metal sample is first pressed into the specified depth of the pit at a certain pressure and maintained for a period of time (generally 30 to 60 s). The load is then removed, and the value of the read notch diameter d and the corresponding depth h is read. According to the above, two values can be converted to the Brinell or Vickers hardness value; it can also be converted to the corresponding Richter, Rockwell or other standard hardness values by the conversion device [20].

In the specific testing process, according to the determined location of the characteristic points, the Leeb hardness tester is used to impact the forging in turn to obtain the measured value of Leeb hardness at the characteristic points [20], and then the ratio of elastic rebound speed and striking speed under the influence of residual stress is obtained. The image acquisition software of the computer and the microscope camera are used to take photographs and measure the indentation size of the feature points. Then, in the CAXA software (2021 version, Beijing Digital Dafang Technology Co., Ltd., Beijing, China), the photos of the feature points and the ruler are imported and combined into an engineering drawing, and the diameter of the indentation is obtained by comparing the ruler, so as to calculate the actual diameter of the indentation (the average value of the three directions) and the indentation depth. These parameters are the basic parameters of the residual stress characterization of forging [21].

The residual stress of forgings is characterized by the apparent elastic modulus affected by the residual stress calculated by impact rebound. The specific process is as follows: ratio coefficient of actual indentation depth to elastic rebound depth according to material and state calibration → residual indentation depth iterated according to indentation diameter → rebound depth obtained → then, calculate the rebound curve of impact indentation according to the rebound speed and energy represented by hardness → calculate the apparent elastic modulus affected by residual stress according to the rebound curve and standard algorithm → calculate the apparent elastic modulus → according to the apparent elastic modulus, the residual stress value of forging measurement point is calculated [22].

The Leeb hardness tester can choose a different hardness system, such as: Brinell hardness, Leeb hardness, Vickers hardness, etc. The hardness system used in this test is Leeb hardness [20]. The Leeb hardness tester consists of a host and an impact device. According to the forging material, the estimated residual stress and the surface state of the forging, the Leeb hardness tester shaped impact device G is used in this test. The Leeb Hardness tester G-type impact head is suitable for measuring heavy, rough surfaces, and the residual stress influence of large casting and forging parts. The impact energy of the G-type impact head is 90 mJ, the mass of the impact body is 20.0 g, the diameter of the ball head of indenter is 5 mm, the material of the ball head is tungsten carbide, its Poisson ratio is 0.21, and the Young’s modulus is 710 GPa. The specific parameters are shown in Table 1.

The Leeb hardness tester uses the interaction between the permanent magnet installed on the impact body and the induction coil to measure the speed; that is, when the impact body passes through the magnetic induction coil, the difference between the impact speed and the rebound speed will make the coil produce a small voltage difference. After these small voltage differences are calculated, the Leeb hardness value can be calculated using Equation (23):

(23) $$HL=1000\frac{v_{R}}{v_{A}}$$

where *HL* is the Leeb hardness value; *v*_R_ is the rebound speed of the impact body; *v*_A_ is the impact velocity of the impact body.

Then, the kinetic energy *E*_z_ before impact body and *E_k_* after impact body meet the following formula:

(24) $$\frac{E_{k}}{E_{z}}=\frac{\frac{1}{2}mv_{R}^{2}}{\frac{1}{2}mv_{A}^{2}}=\frac{HL^{2}}{1000000}$$

According to the forging residual stress formation mechanism and process control mechanism, the residual stress value at each feature point is equivalent to the characteristic temperature value (residual stress converted by thermal stress) or the characteristic load value (residual stress converted by deformation). At the same time, the residual stress contour line of the three-dimensional contour surface is fitted by mathematical software. Based on this, the three-dimensional distribution of residual stress in the thickness dimension of forging is expanded, and the heat or force analysis and calculation module in the finite element software is used to calculate the contour surface field quantity to the three-dimensional solid field quantity. Finally, the result is drawn in the form of a three-dimensional solid cloud image by using the software-defined state variable. The distribution of residual stress can be characterized by animation expression and the subdivision of typical cross sections or truncated lines [21].

#### 2.2.2. Borehole Stress Release Method

**Figure 5:**
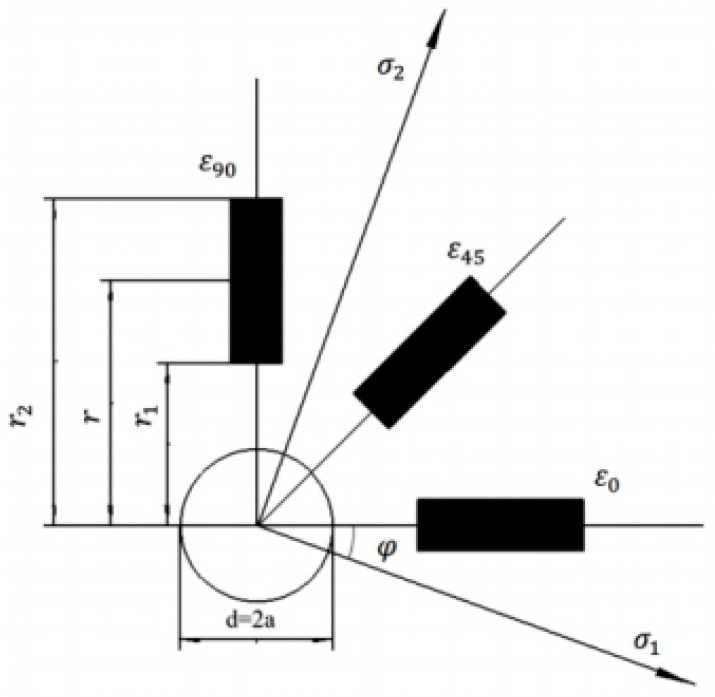
Schematic diagram of residual stress measurement by strain gage hole drilling method.

The schematic diagram of residual stress measurement by the drilling stress release method is shown in Figure 5 [23]. A small hole with a diameter of 1–4 mm is drilled on the part with residual stress, three strain gauges are pasted around the hole at a specified angle, and the angle between the strain gauges and the transverse is 0°, 45° and 90°, respectively, so as to obtain the material strain changes of the parts, caused by the drilling residual stress release. The basic principle is: if there is a self-balanced residual stress in the mechanical parts, when a blind hole of a certain length is drilled at any position in its stress field, the residual stress balance there will change, and the material around the hole will release the corresponding displacement and strain, until a new equilibrium stress field with zero stress in the hole is formed. Based on the strain values released around the small hole measured by the three strain gauges, the original residual stress magnitude and direction can be determined by converting them.

As shown in Figure 5, additional parameters can be further determined by the magnitude of the stress at 0°, 45° and 90° angles:

(25) $$\sigma_{1}=\frac{\varepsilon_{0}+\varepsilon_{90}}{4A}-\frac{1}{4B}\sqrt{{\left(\varepsilon_{0}-\varepsilon_{90}\right)}^{2}+{\left(2\varepsilon_{45}-\varepsilon_{0}-\varepsilon_{90}\right)}^{2}}$$

(26) $$\sigma_{1}=\frac{\varepsilon_{0}+\varepsilon_{90}}{4A}+\frac{1}{4B}\sqrt{{\left(\varepsilon_{0}-\varepsilon_{90}\right)}^{2}+{\left(2\varepsilon_{45}-\varepsilon_{0}-\varepsilon_{90}\right)}^{2}}$$

(27) $$\tan2\varphi =\frac{2\varepsilon_{45}-\varepsilon_{0}-\varepsilon_{90}}{\varepsilon_{90}-\varepsilon_{0}}$$

where, *σ*_1_ is the maximum residual principal stress; *σ*_2_ is the minimum residual principal stress; *φ* is the angle between the residual principal stress *σ*_1_ and the zero-axis sensor; and *A* and *B* are strain release coefficients, which can be obtained by Kirsch theory or calibrated by experiment as follows:

(28) $$A=\left(-\frac{1+v}{2E}\right)\frac{d^{2}}{4r_{1}r_{2}}$$

(29) $$B=-\frac{d^{2}}{2Er_{1}r_{2}}\left[1-\left(\frac{1+v}{4}\right)\frac{d^{2}(r_{1}^{2}+r_{1}r_{2}+r_{2}^{2})}{4r_{1}^{2}r_{2}^{2}}\right]$$

### 2.3. Analytical Method

#### 2.3.1. Dimensionless Analysis

Dimensionless analysis is a method of analyzing natural phenomena and engineering problems through the study of the relationship between physical quantities. If some physical quantities have their specified units of measurement, the units of measurement of other physical quantities will also be determined. In general mechanical problems, any physical quantity *X* can be expressed by the relation of the three basic dimensions of length, mass and time [24,25].

The theoretical core of dimensionless analysis is the Π theorem [26] proposed by E. Buckingham, which states:

(30) $$X=L^{\alpha }M^{\beta }T^{\gamma }$$

For a certain class of physics problem, if there are *n* independent variables *a*_1_, *a*_2_, ⋯, *a_n_*, then the dependent variable *a* can be represented by these *n* independent variables, namely:

(31) $$a=f(a_{1},a_{2}\cdots ,a_{n})$$

If there are *k* independent dimensions in these variables, then the remaining *n − k* dimensions can be expressed as the power of these *k* independent dimensions, and the dimension of the dependent variable *a* can also be expressed by these *k* independent dimensions:

(32) $$\left[a\right]=A_{1}^{m_{1}}A_{2}^{m_{2}}\ldots A_{k}^{m_{k}}$$

Therefore, the variables with their own dimensions in the indentation test can be combined into a dimensionless form, and then the function fitting can be carried out to analyze the law between them. The parameters of this paper are combined according to the Π theorem in dimensional analysis, as shown in Table 2.

#### 2.3.2. Orthogonal Design

Through a few representative experiments or analysis, we can obtain a more comprehensive law of each factor’s influence on the target, and can reasonably know the primary and secondary order of each factor. Therefore, the orthogonal method is used to design the simulation scheme, and the influence of the universal material yield strength, elastic modulus, work hardening index, density, friction coefficient and Poisson’s ratio on the simulation analysis results is discussed. A total of 25 experiments will be tested. This study mainly focuses on the determination of residual stress of metal materials, and the material parameters are selected from the parameter range of common metal materials, such as Young’s modulus, ranging from 40–400 GPa. Table 3 shows the factors and levels of orthogonal design for virtual materials based on typical material combinations.

#### 2.3.3. Inversion Verification

Based on the fitting of dimensionless function and the numerical analysis of *P*–*h* curve, the impact indentation inversion algorithm of material parameters is constructed. In the calculation process, compared with the traditional analysis, the exponential terms in Formulas (9) and (10) are considered as variables; that is, *α* and *β*, as the exponential terms of impact indentation load–displacement response, should be non-scalar terms. According to the simulation calculation of this paper, it is reasonable to determine that *α* and *β* have a certain relationship with the work hardening index *n*.

(33) $$\alpha =0.96159+1.25198n-3.79776n^{2}+6.11217n^{3}$$

(34) $$\frac{\beta }{\alpha }=1.6445-5.78327n+25.39453n^{2}-36.78546n^{3}$$

Using the test measurable values of indentation radius *a*_c_, rebound kinetic energy *E*_k_, and total energy *E*_t_ and Poisson’s ratio of the material as inputs in 25 sets of the virtual material finite element simulation results of orthogonal analysis, we constructed an inversion algorithm flow of the material parameters’ characterization of impact indentation, which is used to analyze the relationship between indentation coefficients (*α*, *β*, etc.) of *P*–*h* curve and the elasto–plastic parameters of the material and apply it to the inversion of the elasto–plastic parameters of the material in real engineering, and subsequently use the apparent elastic modulus E* in the calculation of the residual stresses.

### 2.4. Finite Element Simulation

#### 2.4.1. Finite Element Simulation Software

Abaqus/explicit ABAQUS software (2021 version, Dassault AG, Paris, France.) used in this paper is a kind of engineering simulation finite element software, which is very powerful and has excellent simulation computing ability. Abaqus/explicit, as an analysis module of ABAQUS, can simulate short and instantaneous dynamic problems, and analyze the stress–displacement relationship, the impact of objects, and other very discontinuous problems. It is very suitable for solving dynamic problems, divided into many time incremental steps to improve accuracy according to engineering needs.

The factors and parameters in the orthogonal design in Section 2.3.2 are selected for virtual material parameters and properties. 

**Figure 6:**
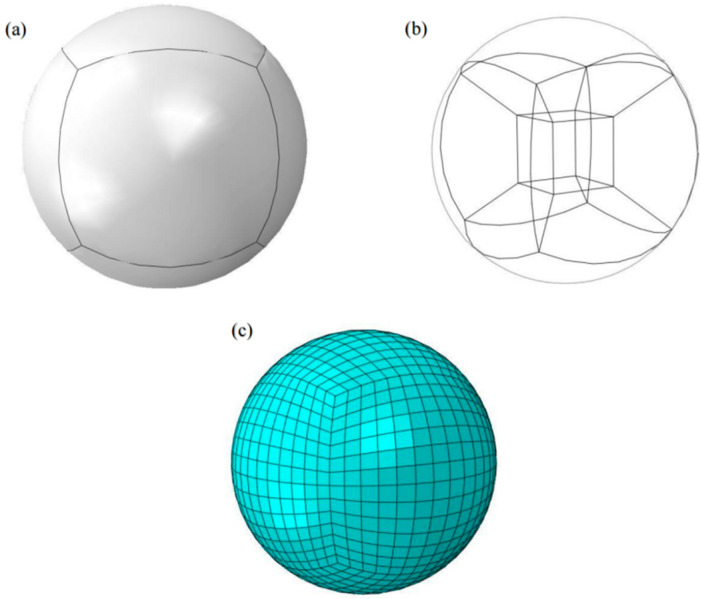
Schematic diagram of impact body model: (**a**) appearance; (**b**) wireframe; (**c**) grid.

**Figure 7:**
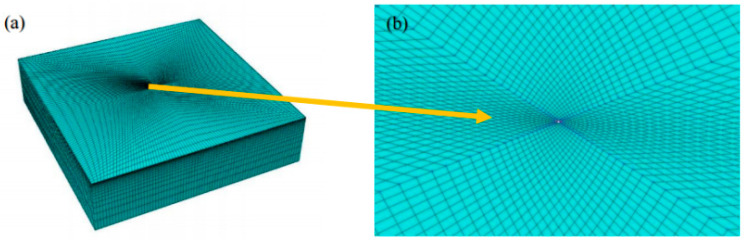
Meshing of the specimen: (**a**) overall mesh map; (**b**) detail of the central area.

In the analysis, the impact head was simplified into a ball with a radius of 2.5 mm, and was subdivided into 7 parts for the ball with mesh rules. The mesh seed density was 0.15 mm, and the elastic impact head used a C3D8Relement. C3D8R elements were used for the samples subjected to impact deformation, and local mesh refinement was required for the central contact area, as shown in Figure 6 and Figure 7. If the rigid impact head is simplified, R3D4 element is used, as shown in Figure 7a.

In order to calculate the interaction between the impact head and the material, it is necessary to define the contact between them; the contact is defined as follows.

The surface is used to transmit normal and tangential forces. The spherical surface of the impact head is defined as the main surface, and the node set in the central area of the specimen is the slave surface. The unidirectional erosion behavior of the impact head on the specimen is simulated.

**Figure 8:**
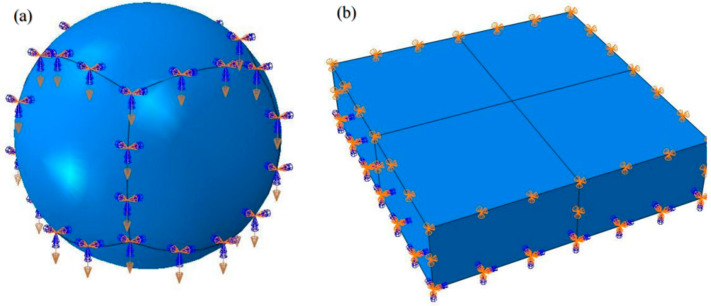
Boundary conditions and load settings: (**a**) punch speed constraint; (**b**) specimen displacement constraint.

The spherical surface of the impact head is defined as the main surface, and the node set in the central area of the specimen is the slave surface. The unidirectional erosion behavior of the impact head on the specimen is simulated, as shown in Figure 8.

#### 2.4.2. Dynamic Analysis Method

The dynamic analysis method is the process of solving equations of motion, geometric equations and physical equations of a given body under its boundary conditions, mainly studying the relationship between the forces applied to the body and the motion of the body. According to the finite element theory, the solution equation can be obtained:

(35) $$M\overset{˙˙}{u}+C\dot{u}+Ku=P$$

where *M* is the structure mass matrix; *C* is the structure damping matrix; *K* is the structural stiffness matrix; *P* is the external force load that changes with time; *u* is the node displacement vector; $\dot{u}$ is the node velocity vector; *ü* is the node acceleration vector.

### 2.5. Technical Route

**Figure 9:**
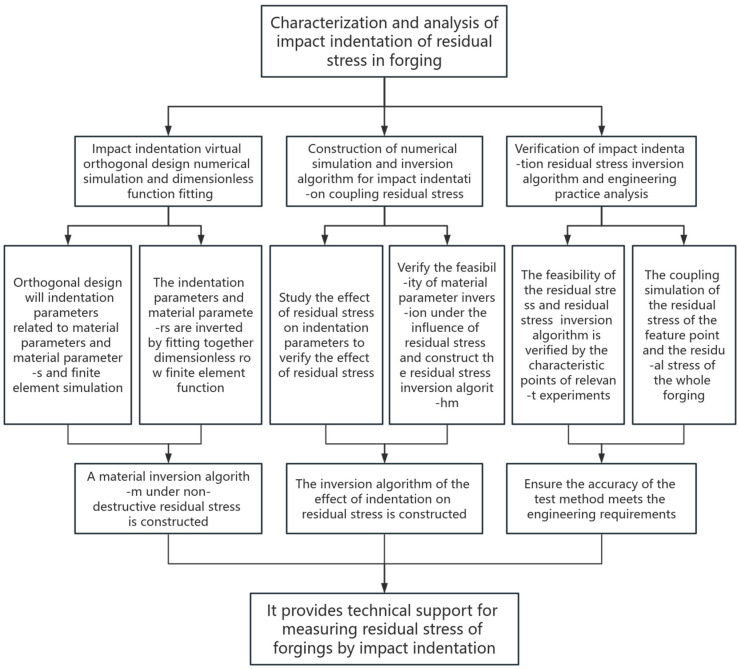
The realization path of the technical route.

The following flow chart (Figure 9) is the realization path of the technical route.

## 3. Results and Discussion

### 3.1. Impact Indentation Simulation Analysis

#### 3.1.1. Simulated Orthogonal Analysis

The main indentation parameters include maximum indentation load *P*_m_, rebound kinetic energy *E*_k_, maximum indentation depth *h*_m_, residual indentation depth *h*_r_, and indentation contact radius *a*_c_. The results in Table 4 are obtained by the range analysis of these parameters; ($\bar{K_{ij}}$ is the average value under each factor level, *R*_i_ is the range of each factor).

**Figure 10:**
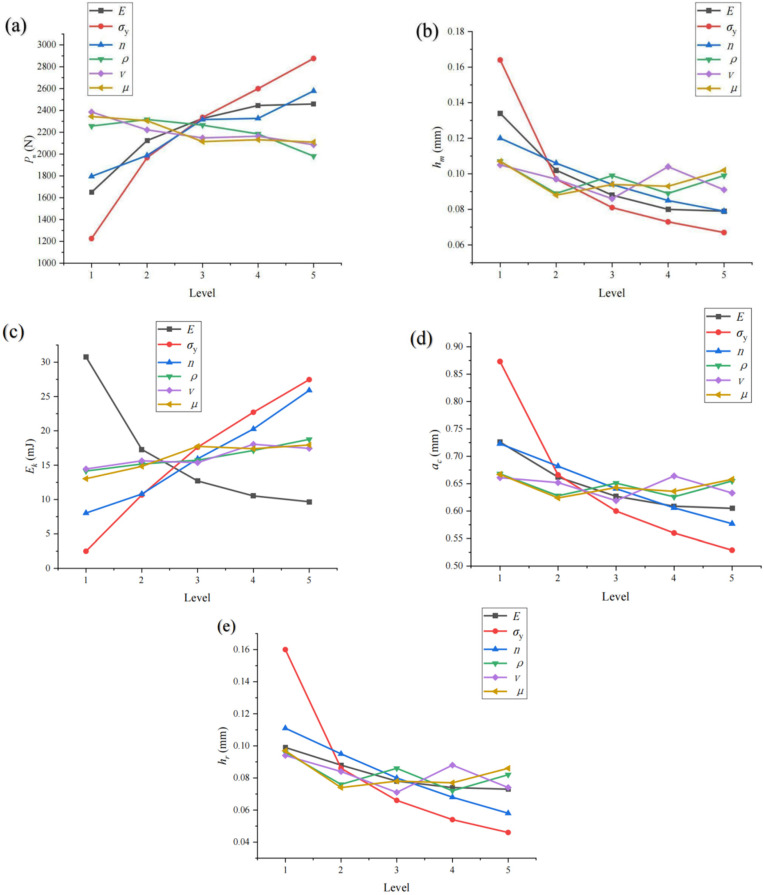
Relationship between indicators and factor levels: (**a**) *P*_m_; (**b**) *h*_m_; (**c**) *E*_k_; (**d**) *a*_c_; (**e**) *h*_r_.

According to the relationship between the $\bar{K_{ij}}$ value under each factor level and the five indicators in the indentation parameters, scatter line charts were each drawn for analysis, as shown in Figure 10. The range value *R*_i_ can show the influence range of these six material parameters on the five indentation parameters, and the standard error *SE*_i_ can show different degrees of influence. Combined with the range value *R*_i_ and standard error *SE*_i_ in Table 4, it can be judged that the three significant factors that have the greatest influence on the selected indexes in the indentation parameters are yield strength *σ*_y_, elastic modulus *E* and work hardening index *n*, and their influence rules are listed in Table 5 (↑ indicates increase, ↓ indicates decrease).

#### 3.1.2. Dimensionless Impact Indentation Analysis

**Figure 11:**
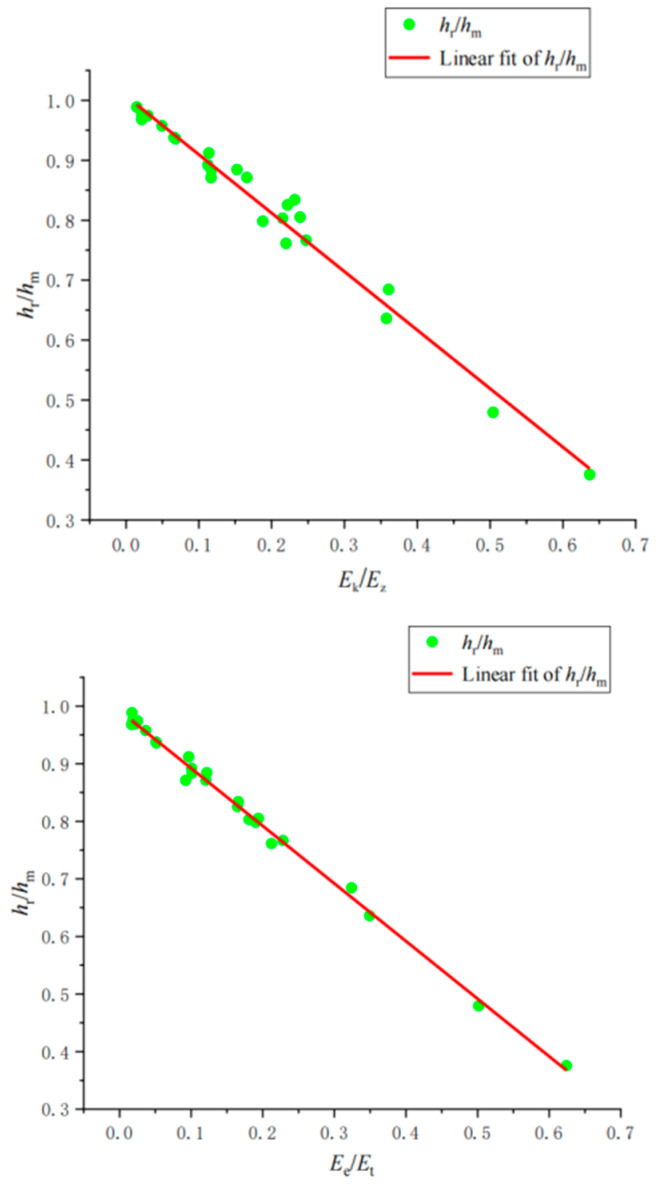
Fitting law of *h*_r_/*h*_m_, *E*_k_/*E*_z_ and *E*_e_/*E*_t_.

Based on the extraction of the main influencing factors on materials, the indentation parameters and dimensionless parameters are combined, and the mutual influence and change rule of the material dimensionless parameters on the indentation dimensionless parameters are studied. Through SPSS bivariate analysis, it is found that the Pearson coefficient between *h*_r_/*h*_m_ and *E*_k_/*E*_z_ is −0.993, and the Pearson coefficient between *h*_r_/*h*_m_ and *E*_e_/*E*_t_ is −0.999, indicating that *h*_r_/*h*_m_ has a significant linear relationship with *E*_k_/*E*_z_ and *E*_e_/*E*_t_. The relationships between *h*_r_/*h*_m_ and *E*_k_/*E*_z_ and *h*_r_/*h*_m_ and *E*_e_/*E*_t_ can be fitted with linear relationships in Figure 11:

(36) $$\frac{h_{r}}{h_{m}}=0.99206-1.00066\frac{E_{e}}{E_{t}}$$

(37) $$\frac{h_{r}}{h_{m}}=1.00717-0.97625\frac{E_{k}}{E_{z}}$$

**Figure 12:**
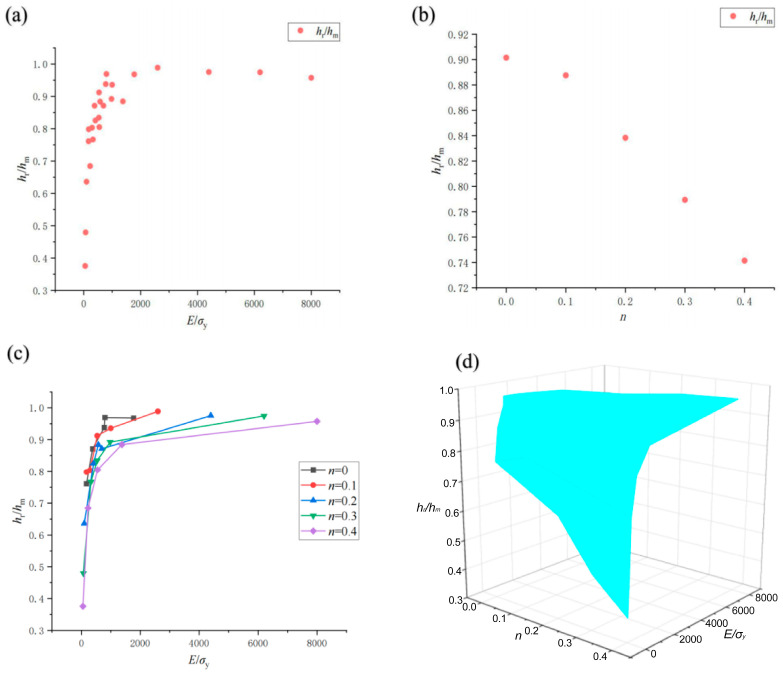
Schematic diagram of the change rule of *h*_r_/*h*_m_ of 25 groups of virtual materials with *E*/*σ*_y_ and *n* (**a**) *h*_r_/*h*_m_ and *E*/*σ*_y_; (**b**) *h*_r_/*h*_m_ and *n*; (**c**) *h*_r_/*h*_m_ and *E*/*σ*_y_ with different *n*; (**d**) 3D surface (*h*_r_/*h*_m_ with *E*/*σ*_y_ and *n*).

**Figure 13:**
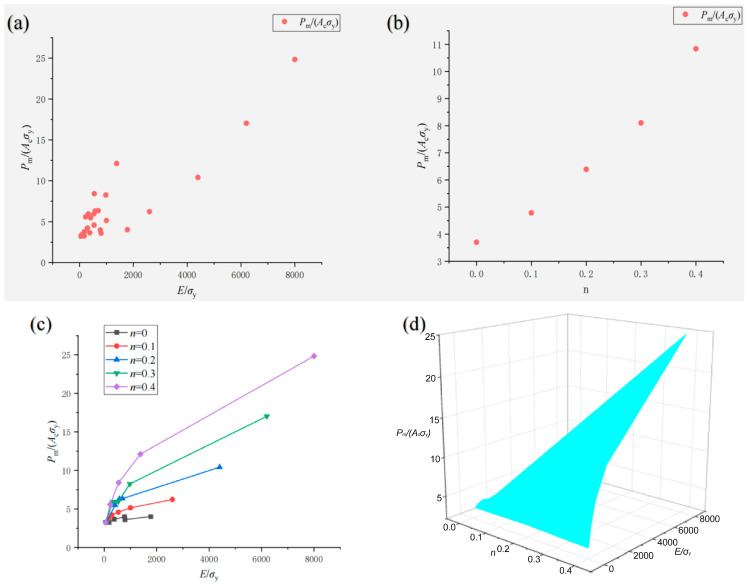
Schematic diagram of the change rule of *P*_m_/(*A*_c_*σ*_y_) of 25 groups of virtual materials with *E*/*σ*_y_ and *n* (**a**) *P*_m_/(*A*_c_*σ*_y_) and *E*/*σ*_y_; (**b**) *P*_m_/(*A*_c_*σ*_y_)and *n*; (**c**) *P*_m_/(*A*_c_*σ*_y_) and *E*/*σ*_y_ with different *n*; (**d**) 3D surface (*P*_m_/(*A*_c_*σ*_y_) with *E*/*σ*_y_ and *n*).

The effects of material dimensionless parameters *E*/*σ*_y_ and *n* on the dimensionless indentation parameters *h*_r_/*h*_m_ and *P*_m_/(*A*_c_*σ*_y_) are given in Figure 12 and Figure 13. It can be seen that *h*_r_/*h*_m_, *P*_m_/(*A*_c_*σ*_y_) and *E*/*σ*_y_ show obvious logarithmic changes.

From the above research, *h*_r_/*h*_m_ and *P*_m_/(*A*_c_*σ*_y_) change logarithmically with *E*/*σ*_y_ and as a power function with *n*, respectively. Further, this can be generalized to study whether other indentation dimensionless parameters can still be fitted with similar relations. Therefore, SPSS software (Version 2021, SPSS Inc., Chicago, IL, USA) is used to conduct a nonlinear analysis of the indentation dimensionless parameters and the material dimensionless parameters. A residual sum of squares analysis was used to evaluate the degree of fitting between the regression curve and the average law of the original data with *R*^2^ (*R* square coefficient). The closer the value is to 1, the better the fitting effect of the relationship, thus obtaining the following Π function and *R*^2^.

(38) $$\begin{array}{l} \prod_{1}=\frac{E_{k}}{E_{z}}=-0.449075-27.149230n+149.682750n^{2}-155.176579n^{3}\\ +(0.671938+10.938856n-57.589806n^{2}+52.817654n^{3})\ln\left(\frac{E^{*}}{\sigma_{y}}\right)\\ +(-0.159467-1.393058n+7.220232n^{2}-5.601990n^{3}){\left[\ln\left(\frac{E^{*}}{\sigma_{y}}\right)\right]}^{2}\\ +(0.010432+0.055481n-0.294549n^{2}+0.182078n^{3}){\left[\ln\left(\frac{E^{*}}{\sigma_{y}}\right)\right]}^{3} \end{array}$$

(39) $$\begin{array}{l} \prod_{2}=\frac{E_{e}}{E_{t}}=2.809288-22.783795n+133.955533n^{2}-187.65353n^{3}\\ +(0.879374+9.512336n-55.63027n^{2}+78.05191n^{3})\ln\left(\frac{E^{*}}{\sigma_{y}}\right)\\ +(0.082443-1.263785n+7.559037n^{2}-10.62542n^{3}){\left[\ln\left(\frac{E^{*}}{\sigma_{y}}\right)\right]}^{2}\\ +(-0.00197+0.053164n-0.334668n^{2}+0.47153n^{3}){\left[\ln\left(\frac{E^{*}}{\sigma_{y}}\right)\right]}^{3} \end{array}$$

(40) $$\begin{array}{l} \prod_{3}=\frac{h_{r}}{h_{m}}=-1.29512+23.274709n-138.647129n^{2}+190.314256n^{3}\\ +(0.585552-9.582637n+57.811719n^{2}-79.644709n^{3})\ln\left(\frac{E^{*}}{\sigma_{y}}\right)\\ +(-0.031112+1.252762n-7.914212n^{2}+10.970854n^{3})\ln{\left(\frac{E^{*}}{\sigma_{y}}\right)}^{2}\\ +(-0.00089-0.051289n+0.352528n^{2}-0.494537n^{3})\ln{\left(\frac{E^{*}}{\sigma_{y}}\right)}^{3} \end{array}$$

(41) $$\begin{array}{l} \prod_{4}=\frac{P_{m}}{A_{c}\sigma_{y}}=-12.976294-454.561515n+1835.993543n^{2}-1468.556182n^{3}\\ +(6.828175+193.267474n-692.604798n^{2}+400.308668n^{3})\ln\left(\frac{E^{*}}{\sigma_{y}}\right)\\ +(-0.940733-26.675947n+80.094101n^{2}-19.695177n^{3})\ln{\left(\frac{E^{*}}{\sigma_{y}}\right)}^{2}\\ +(0.044216+1.245222n-2.736746n^{2}-0.822512n^{3})\ln{\left(\frac{E^{*}}{\sigma_{y}}\right)}^{3} \end{array}$$

where *E*_z_ is the kinetic energy before the impact of the impact body; *E*_k_ is the kinetic energy after the rebound of the impact body; E* is the reduced modulus, the unit is MPa; *σ*_y_ is the yield strength in MPa; *n* is the work hardening index; *E*_t_ is the total energy of the load–displacement curve; *E*_e_ is the elastic rebound of the material to the impact body; *h*_r_ is the depth of residual indentation; *h*_m_ is the maximum pressing depth; *P*_m_ is the peak load of impact indentation; the projected area of *A*_c_ is the residual indentation; *H*_c_ is defined as the indentation hardness, which can be calculated from the peak load and the projected area of the residual indentation.

After the above non-linear fits, the dimensionless *R*^2^ is all greater than 0.99, that is, the sum of squares of the residuals are all in the order of 10^−3^, and the fitting effect of *R*^2^ is very ideal.

#### 3.1.3. Material Parameter Inversion Verification

**Figure 14:**
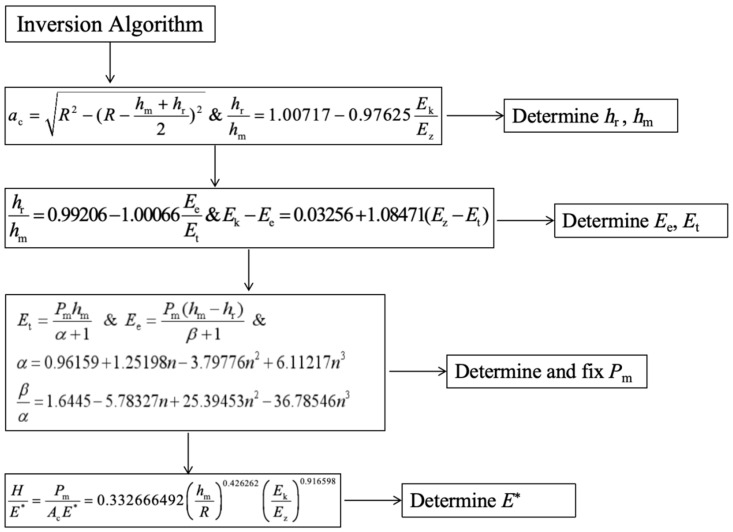
Flowchart of material parameters’ reverse algorithm.

**Figure 15:**
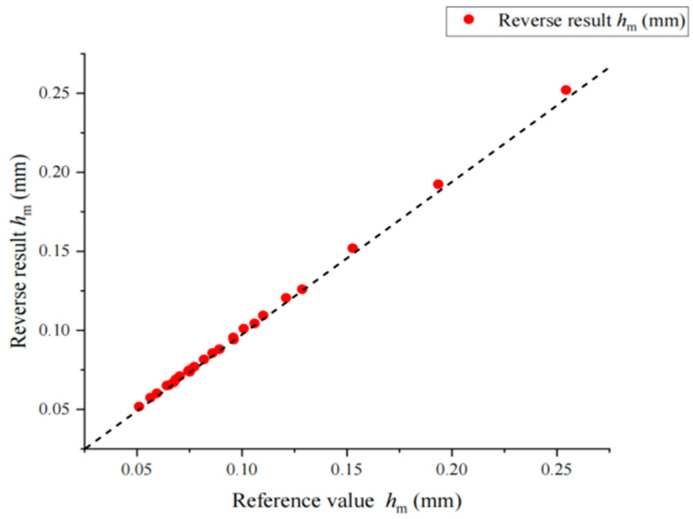
Comparison between *h*_m_ inversion result and reference value. The dashed lines in the figure are 45 degrees diagonals.

**Figure 16:**
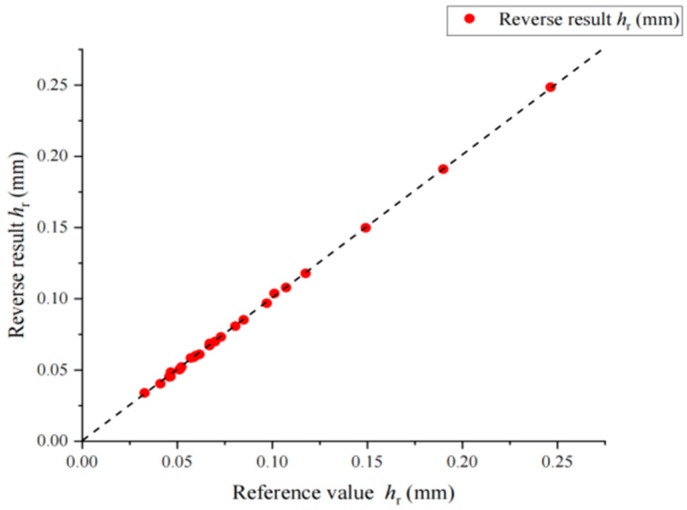
Comparison between *h*_r_ inversion result and reference value. The dashed lines in the figure are 45 degrees diagonals.

**Figure 17:**
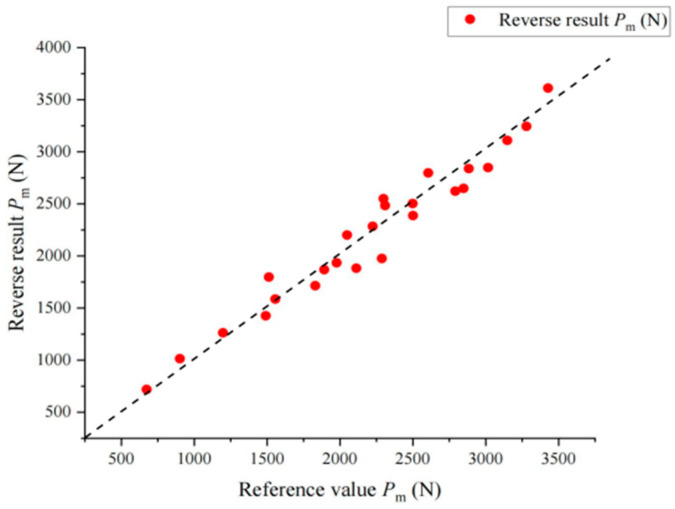
Comparison between *P*_m_ inversion result and reference value. The dashed lines in the figure are 45 degrees diagonals.

After the comparative simulation calculation, inversion analysis is carried out. The flow and formula of inversion calculation are given in Figure 14. The relative error between the inversion values of *h*_m_ and *h*_r_ and the reference values is less than 3%. The relative error of the inversion values of calculated *P*_m_ is less than 5% for most virtual materials. The reduced elastic modulus of the material is obtained by inversion, and then transformed into the elastic modulus of the material through Equation (4). Since the material parameters in the simulation are set by adopting the orthogonal design method, the average value of the elastic modulus with the same reference value is obtained to exclude the influence of other factors on the inversion of the elastic modulus. As shown in Table 6, the error value of the elastic modulus calculated by inversion is controlled within 10%, and the diagrams of the comparison between the reverse results and its corresponding values are shown in Figure 15, Figure 16 and Figure 17.

### 3.2. Impact Indentation Coupled Residual Stress Simulation Analysis

#### 3.2.1. Residual Stress Simulation Results

**Figure 18:**
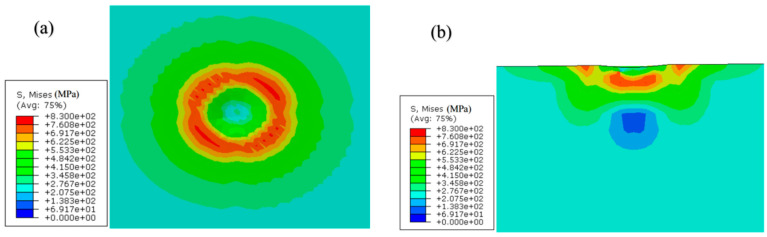
Mises stress nephogram of residual compressive stress specimen after impact (using compression of Mises stress of 243 MPa as an example): (**a**) horizontal plane; (**b**) vertical section.

**Figure 19:**
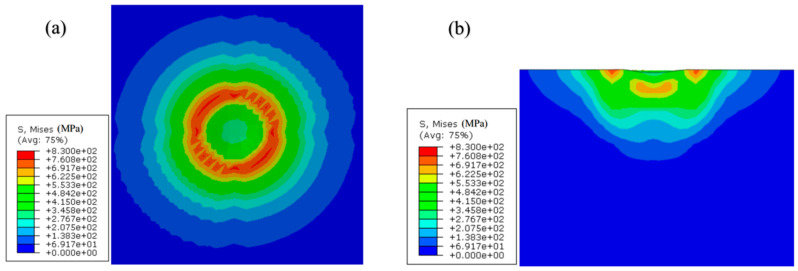
Mises stress nephogram of specimen without residual stress after impact (compare with compression of Mises stress of 243 MPa): (**a**) horizontal plane; (**b**) vertical section.

**Figure 20:**
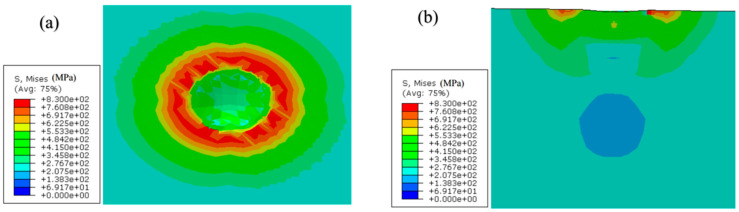
Mises stress nephogram of residual tensile stress specimen after impact (taking the extention of Mises stress of 243 MPa as an example): (**a**) horizontal plane; (**b**) vertical section.

The following Figure 18, Figure 19 and Figure 20 show the Mises stress distribution cloud graph for impact simulation under compressive stress, no stress and tensile stress, respectively. It can be analyzed from these figures that, regardless of the existence of residual stress, the stress state of the material further away from the impact position almost maintains the original stress and stress state after being impacted by the impact body, so it can be considered as equivalent to the semi-infinite body simulation. The research shows that the presence of residual stress will make a local area under the impact position appear after the material bears the impact. Compared with the specimen without residual stress, the residual compressive stress will make the local area under the impact position appear with greater stress, while the residual tensile stress will make the local area under the impact position have relatively lower stress.

#### 3.2.2. Simulated Orthogonal Analysis

The range analysis table of the impact indentation simulation shows the results of the 18 groups of virtual materials with orthogonal design, and by comparing the *R*′_1_, *R*′_2_, …, *R*′_7_, it can be concluded that the top three material parameters that have the greatest influence on impact indentation parameters are still elastic modulus *E*, yield strength *σ*_y_ and work hardening index *n*, among which yield strength *σ*_y_ is still the most significant material parameter, while the influence of other parameters on the indentation process is relatively low. Therefore, it can be concluded that when analyzing the coupling residual stress of materials, the coupling residual stress is still consistent with the analytical results without the influence of residual stress, because this paper conducted an orthogonal analysis based on universal materials and adopted a dimensionless analysis.

As can be seen from Table 7, the influence of *σ*_r_/*σ*_y_ on indentation parameters is not significant, but the quantitative expression of residual stress in *h*_m_, *h*_r_ and *a*_c_ is relatively obvious.

**Figure 21:**
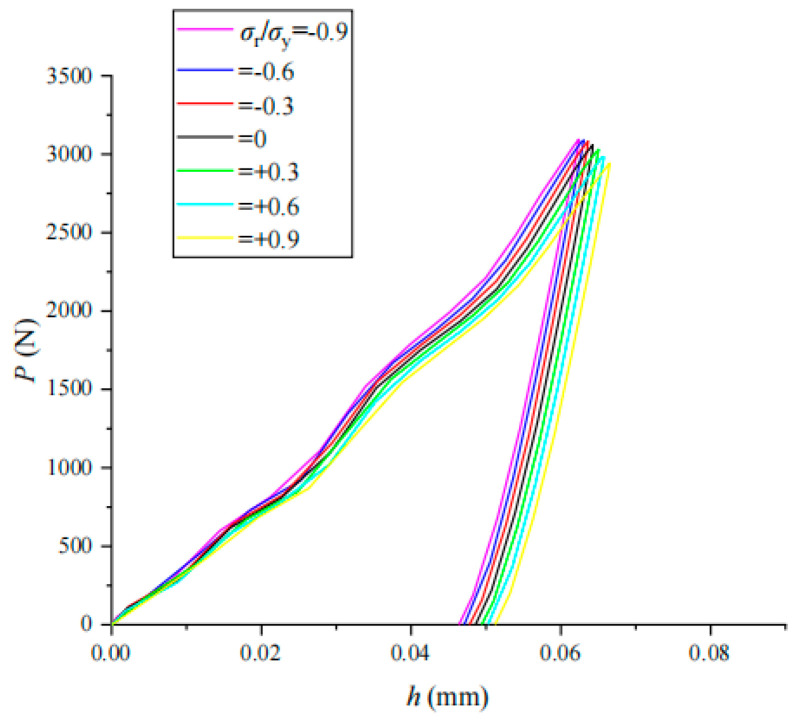
Effect of *σ*_r_/*σ*_y_ on *P*–*h* curve in 304 stainless steel.

**Figure 22:**
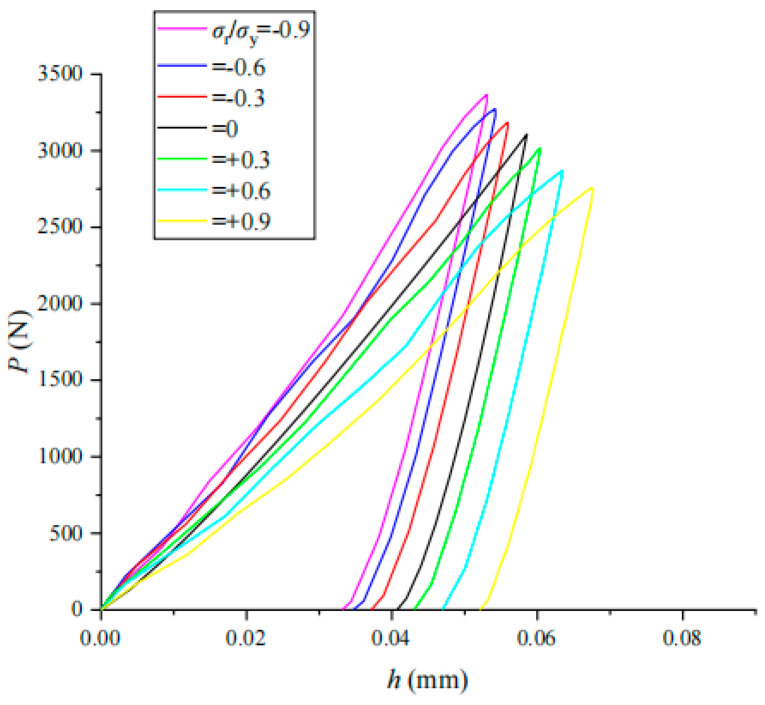
Effect of *σ*_r_/*σ*_y_ on *P*–*h* curve in 35Cr2Ni4MoA.

**Figure 23:**
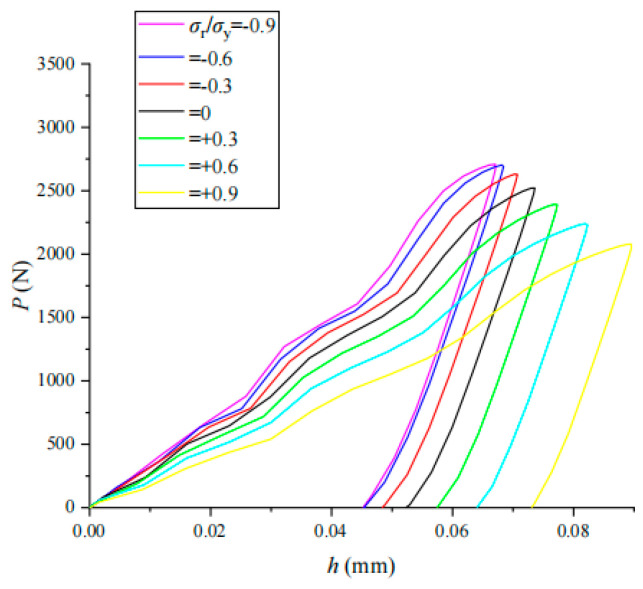
Effect of *σ*_r_/*σ*_y_ on *P*–*h* curve in TC4-DT.

The indentation load–displacement (*P*–*h*) curves of coupling *σ*_r_/*σ*_y_ for three typical materials (304 stainless steel, 35Cr2Ni4MoA alloy steel and TC4-DT titanium alloy) were obtained by an impact indentation simulation of coupling residual stress with different values of *σ*_r_/*σ*_y_ (shown in Figure 21, Figure 22 and Figure 23). The impact indentation curve has an obvious correlation with the residual stress and changes regularly with the magnitude and nature of the residual stress. As the additional tensile residual stress increases, both *h*_m_ and *h*_r_ increase but *P*_m_ decreases. As the compressive residual stress increases, both *h*_m_ and *h*_r_ decrease while *P*_m_ increases.

A comparative analysis of the load–displacement *P*–*h* curves of the three materials shows that the residual stress has relatively little effect on the *P*–*h* curves of 304 stainless steel. The unloading part of the load–displacement curve of each material is almost the same; that is, the change in the initial unloading slope needs to be accurately analyzed with a clear mathematical function form, so the impact of residual stress on the unloading curve of impact indentation is less than that at the loading stage.

**Figure 24:**
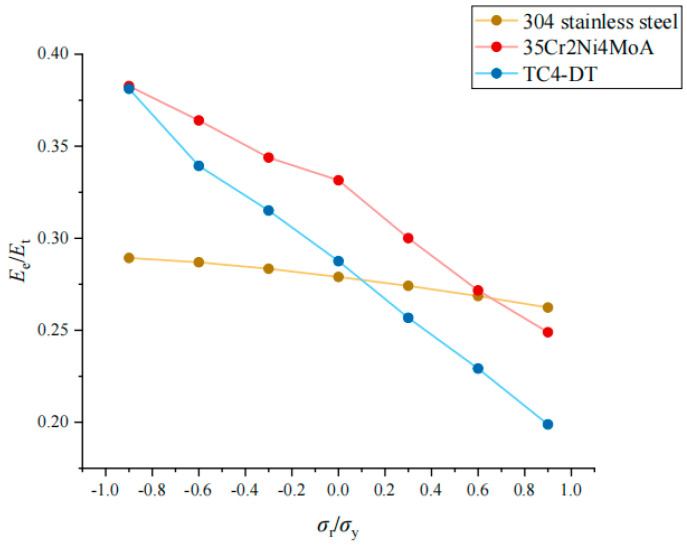
Effect of *σ*_r_/*σ*_y_ on *E*_e_/*E*_t_ under different materials.

The *σ*_r_/*σ*_y_ and *E*_e_/*E*_t_ of each material show a linear relationship, but the influence of *σ*_r_/*σ*_y_ of the three materials on *E*_e_/*E*_t_ is different. The greater the E*/*σ*_y_ is, the greater the influence of *σ*_r_/*σ*_y_ on *E*_e_/*E*_t_ is, as shown in Figure 24.

#### 3.2.3. Residual Stress Inversion Verification

**Figure 25:**
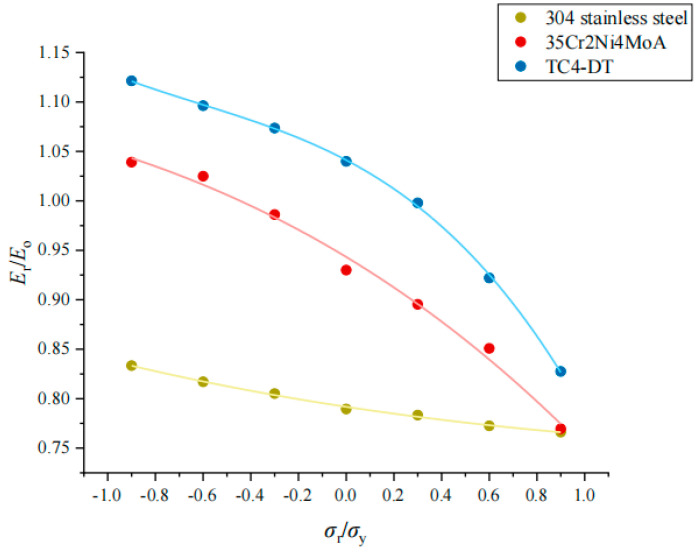
The relationship of *E*_r_/*E*_o_ and *σ*_r_/*σ*_y_ in three materials.

The intrinsic modulus of the material can be calculated from the previous inversion process. In the coupled residual stress analysis, the ratio of the apparent elastic modulus *E*_r_ to the material elastic modulus *E*_o_ under the influence of the residual stress (*E*_r_/*E*_o_) and its relationship to *σ*_r_/*σ*_y_ can also be obtained, as shown in Figure 25. Then, the calculation results of fitting coefficients of dimensionless functions for the three materials are shown in Table 8.

(42) $$\frac{E_{r}}{E_{0}}=\sum_{i=0}^{3}c_{i}{\left(\frac{\sigma_{r}}{\sigma_{y}}\right)}^{i}$$

#### 3.2.4. Borehole Stress Release Method

**Figure 26:**
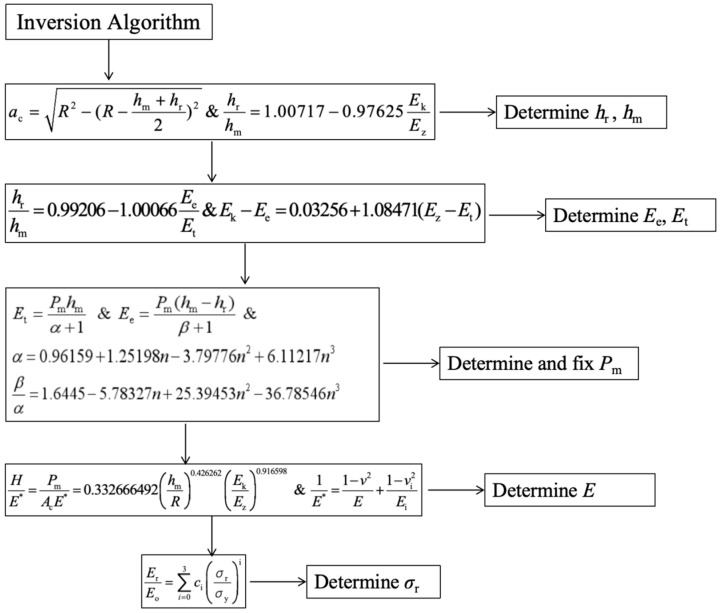
Flow chart of inversion algorithm for residual stress.

**Figure 27:**
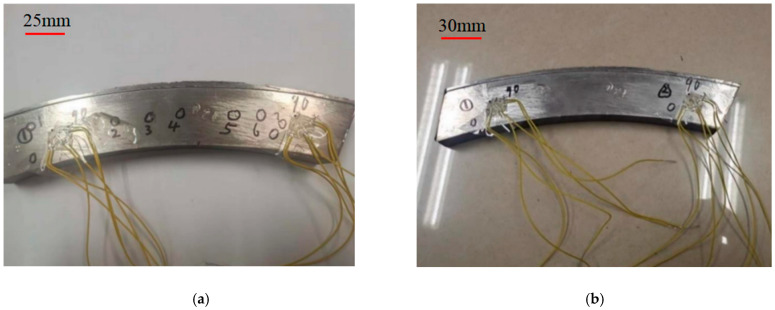
(**a**) Schematic diagram of measuring points of impact indentation for TC4-DT titanium alloy tested forging; (**b**) Point layout of the stress relieving drilling method for residual stress measurement.

The impact indenter used in the test is a Leeb hardness tester. The accuracy of this algorithm can be further improved through the inversion calculation flow shown in Figure 26. For comparative analysis, 7 locations were tested, as shown in Figure 27, and the indentation radius of each location was measured with a D30 optical microscope. Finally, the residual stress *σ*_r_ at each point is calculated, as shown in Table 9.

Figure 27b shows the schematic diagram of testing residual stress by the borehole stress release method. The strain release results measured at two different positions of TC4-DT titanium alloy tested forging are shown in Table 10. The residual stress calculation Formulas (25)–(27) of the strain flower, as shown in Figure 5, were used to analyze and calculate each measuring point, and the residual stress magnitude of the two measuring points could be given, as shown in Table 10.

The residual tensile stress at the left end of the specimen is smaller than the residual tensile stress at the right end, and the values of the nearest neighbor points are consistent. As can be seen from Figure 27a, the positions of measurement points 1 and ① are close to each other, and the difference between the residual stress inversion and the test is 15.47 MPa, and the relative error is 7.4%. The position of measurement point 7 is close to that of point ②, the difference between residual stress inversion and the test is −21.59 MPa, and the relative error is −6.5%. Therefore, the accuracy of the inversion algorithm of the residual stress impact indentation based on the Leeb hardness tester as the impact tool is proven, and the inversion algorithm of residual stress measurement based on impact indentation can be used in engineering analysis.

### 3.3. Engineering Practice and Analysis of Impact Indentation Characterization of Residual Stress

**Figure 28:**
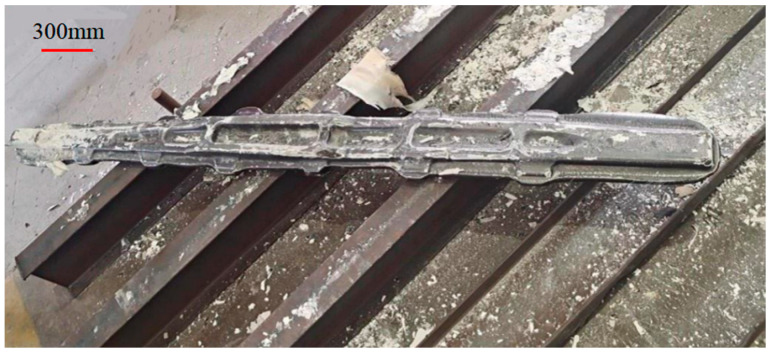
TC4-DT aviation large forgings physical photo.

**Figure 29:**
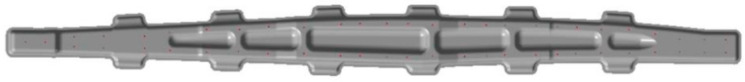
Mathematical model diagram of TC4-DT aviation large forging.

Combined with the engineering application, the residual stress test was carried out for the warping of TC4-DT aviation large forgings. As shown in Figure 28, the length of selected forgings was 2080 mm and the maximum width was 195 mm. To construct a 3D residual stress distribution diagram of forging and facilitate subsequent analysis, it is necessary to select the location of residual stress test feature points reasonably, according to forging geometry, the process path, and the workpiece warping caused by subsequent machining. According to the shape structure of the forging, more feature points are arranged on its front (as shown in Figure 28), while relatively few feature points are arranged on its back. To characterize the three-dimensional residual stress distribution from the surface to the inside of large forgings according to the formation mechanism of residual stress and process control mechanism, the numerical model shown in Figure 29 is used for analysis. The red dot in the figure represents the location of feature points, and about 70 feature points are selected for each forging for on-site measurement.

#### Results Analysis of Residual Stress in Forging

The maximum and minimum values of residual maximum principal stress, residual intermediate principal stress, residual minimum principal stress, residual equivalent stress, residual shear stress and total deformation caused by residual stress of the entire forging were obtained through test analysis, finite element simulation and three-dimensional fitting, as shown in Table 11.

**Figure 30:**
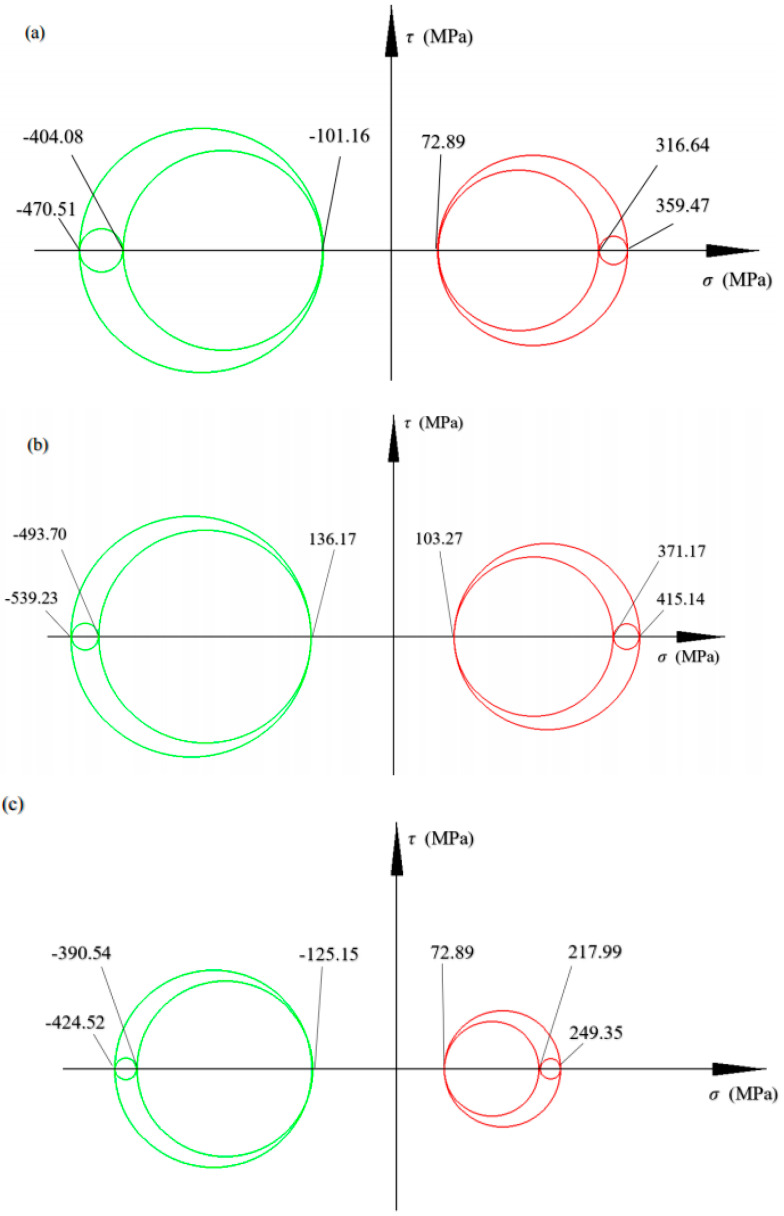
Mohr circle of residual stress state of TC4-DT forgings under different conditions: (**a**) die forgings; (**b**) heat treated forgings (quenching); (**c**) secondary annealed forgings.

To further analyze the residual stress distribution of the whole forging, the maximum and minimum values of the principal stress in the whole forging residual stress under different states are extracted and plotted as a similar stress Mohr circle for analysis (as shown in Figure 30).

To carry out an objective quantitative evaluation of the overall situation of the forging, the maximum and minimum values of the residual principal stress are substituted into the formula:

(43) $$Me=\frac{\sigma_{\max1}+\sigma_{\min3}}{2}$$

(44) $$De=\frac{\sigma_{\max1}-\sigma_{\min3}}{2}$$

(45) $$Eq=\frac{1}{6}{\left({\left(\frac{\sigma_{\max1}-\sigma_{\max2}}{2}\right)}^{2}+{\left(\frac{\sigma_{\max1}-\sigma_{\max3}}{2}\right)}^{2}+{\left(\frac{\sigma_{\max2}-\sigma_{\max3}}{2}\right)}^{2}+{\left(\frac{\sigma_{\min1}-\sigma_{\min2}}{2}\right)}^{2}+{\left(\frac{\sigma_{\min1}-\sigma_{\min3}}{2}\right)}^{2}+{\left(\frac{\sigma_{\min2}-\sigma_{\min3}}{2}\right)}^{2}\right)}^{\frac{1}{2}}$$

where *Me* is the mean square value; *De* is the difference; *Eq* is the equivalent value; and *σ*_max1_, *σ*_max2_ and *σ*_max3_ are the maximum values of maximum residual principal stress, residual intermediate principal stress and minimum residual principal stress of the whole forging, respectively. σ_min1_, *σ*_min2_ and *σ*_min3_ are the minimum values of the maximum residual principal stress, residual intermediate principal stress and residual minimum principal stress of the whole forging, respectively.

The mean *Me* is the average value of the maximum residual principal stress and the minimum residual principal stress, which is used to measure and compare the residual stress of the whole forging. Compared with the residual compressive stress, the residual tensile stress is more harmful to the forging. Therefore, when the mean *Me* is the compressive stress, it means that most areas of the forging are in the residual compressive stress state, and the influence of the residual stress on the forging is relatively low or beneficial. The difference *De* is half of the difference between the maximum residual principal stress and the minimum residual principal stress, and the smaller the value, the smaller the range of residual stress variation in each part of the forging, so the residual stress distribution of the forging is better. When the mean value *Me* is equal, the greater the difference *De*, the greater the difference in residual stress between various parts of the forging, and the easier it is to cause deformation in subsequent processing, which can be used to characterize the degree of deformation in subsequent processing. Equivalent value *Eq* is a representation formula of the overall residual stress of forging derived from the fourth strength theory. When the mean value *Me* and the difference *De* are equal, the smaller the equivalent value *Eq* is, the smaller the shear stress of each part of the forging, and the smaller the distortion degree caused by the residual stress of the forging itself, which can be used to characterize the current deformation caused by the residual stress. According to the overall evaluation of the forging, the deformation caused by residual stress after stress relief annealing is better than that after high temperature die forging, and the state after high temperature die forging is better than that after conventional heat treatment.

**Figure 31:**
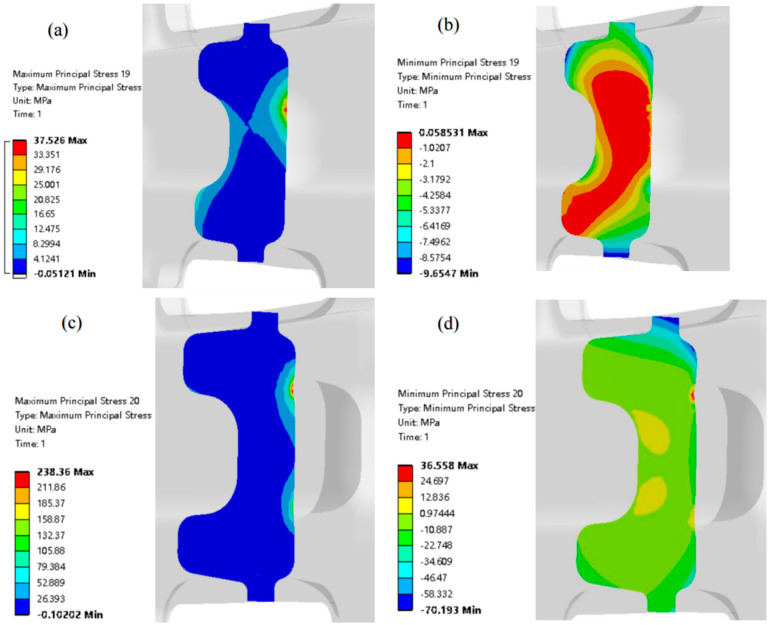
TC4-DT forging typical characteristic section: (**a**) maximum residual principal stress of profile 1; (**b**) residual minimum principal stress of profile 1; (**c**) maximum residual principal stress of profile 2; (**d**) minimum residual principal stress of profile 2.

Figure 31 is the nephogram of the maximum residual principal stress and minimum residual principal stress of the sections of different characteristic parts of TC4-DT die forging. It can be seen from the analysis of Figure 31 that the residual stress value distributed on the overall surface and near surface of the die forging is relatively large, while the residual stress value deep inside the die forging is not large.

**Figure 32:**
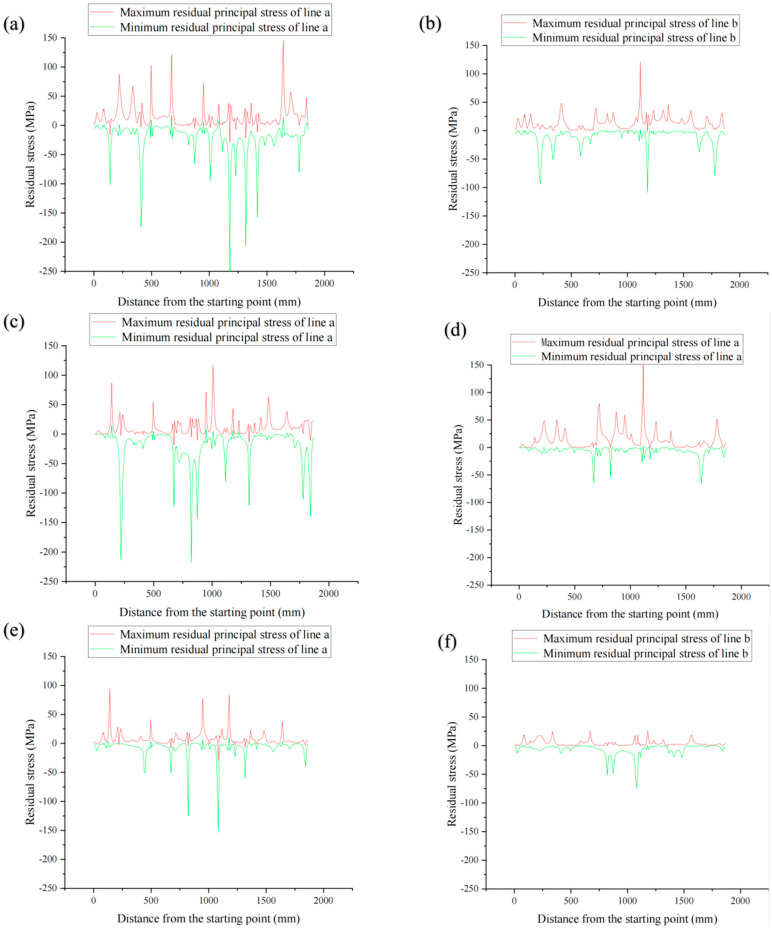
Diagram of residual maximum principal stress and residual minimum principal stress of TC4-DT upper surface line in different states: (**a**) line *a* of die forgings; (**b**) line *b* of die forgings; (**c**) line *a* of heat treated forgings; (**d**) line *b* of heat treated forgings; (**e**) line *a* of annealed forgings; (**f**) line *b* of annealed forgings.

Figure 32 below shows the curves of the maximum residual principal stress and minimum residual principal stress of the connection of the outer contour or near surface feature points of TC4-DT forging. Line *a* and line *b* are the lines of the two sides of the upper surface of the forging, respectively. Based on the third strength theory, this paper determines the possible deformation caused by residual stress at this point by judging the same point difference between the maximum and minimum principal stresses of residual stress on the line. The greater the difference, the greater the possibility of deformation.

The dislocation degree of the maximum principal stress and the minimum principal stress peak (crest and trough) reflects the degree of residual stress change, which can be used to analyze the main cause of residual stress and the elimination method. According to the comprehensive analysis, in the same state of TC4-DT forging, the part of the connection a side is more likely to be deformed and the degree of residual stress is greater than that of the part of the connection b side. For TC4-DT forgings in different states, the deformation caused by residual stress at the forging line after stress removal annealing is the smallest, and the degree of residual stress change is also the smallest. The degree of warping deformation that may be caused by forging can be analyzed by establishing the connection diagram of the characteristic part of forging.

## 4. Conclusions

We have studied and controlled the residual stress of forgings in depth, and, combined with the special needs of aerospace forgings, carried out research on the impact indentation residual stress test, as well as the characterization and engineering practices based on the Leeb hardness tester, providing a new and effective technical route for the characterization and control of residual stress of forgings.

(1) An orthogonal design, combined with finite element simulation, was used to simulate the impact indentation process of a virtual universal material. Using the explicit dynamics module of ABAQUS software, the deformation distribution and stress–strain law during the impact indentation process were analyzed in detail, which verified the reliability of the constructed impact indentation finite element analysis model and confirmed the validity of the power–law principal relationship as the main model of the universal material.
(2) On this basis, the influence law of material parameters and the primary and secondary influence order of material parameters in the impact indentation process were further investigated. It was found that materials with a lower modulus of elasticity accumulate more energy during impact indentation, while an increase in yield strength reduces the accumulated energy of deformation of the material. The higher the work hardening index *n*, the greater the ability of the material to resist deformation. In addition, significant linear relationships between *h*_r_/*h*_m_ and *E*_k_/*E*_t_, *h*_r_/*h*_m_ and *E*_e_/*E*_t_, etc., as well as the logarithmic law of change in *P*_m_/(*A*_c_*σ*_y_) and *E*/*σ*_y_, the law of the power function with *n*, etc., were found.
(3) The obvious correlation between impact indentation curve and residual stress provides a new method to characterize residual stress by impact indentation test. Simulation calculations and experimental and practical demonstrations show that the Leeb hardness tester impact test method has high accuracy and reliability. The calculated values of the indentation curves *h*_m_ and *h*_r_ have a relative error of no more than 3% with respect to the reference values, and the corrected *P*_m_ inversion values for most of the virtual materials have a relative error of no more than 5%. For the folded modulus of elasticity and apparent modulus of elasticity of the materials derived from the inversion, the error values were controlled within 10%. The TC4-DT specimens were analytically characterized for residual stresses, and the errors were also controlled within 10%, which meets the requirements for the engineering testing of residual stresses.

The practical significance of this research is that it not only helps us to deeply understand the formation mechanism of residual stress in aerospace forgings, but also provides us with an effective means of residual stress control. By optimizing the manufacturing process and reducing the level of residual stress, we can further improve the mechanical properties and service life of forgings, thus guaranteeing the safety and reliability of aerospace spacecraft.

Looking to the future, with the continuous development of aerospace technology, the requirements for forgings performance will also be higher and higher. Therefore, the research and control of residual stress will become a focus of continuous attention. We look forward to further research to explore more advanced residual stress testing and characterization methods to provide strong support for the sustainable development of the aerospace industry.

Finally, we suggest that aerospace enterprises, scientific research institutions, universities and other relevant units strengthen the research and application of residual stress testing and control technology. Through the introduction of advanced test equipment and technical means, combined with specific engineering practice, the control level of residual stress is constantly improved, contributing to the prosperity and development of the aerospace industry.

## Figures and Tables

**Table 1:** Parameters of G-type impact device of Leeb hardness tester.

Correlation Parameter	G-Type
Sample reference maximum hardness	650 HB
The average surface roughness of the sample *R*_a_	6.3 μm
Minimum mass of specimen	>15 kg
Need stable support	5~15 kg
Minimum thickness of specimen (stable support can be broken)	10 mm
Minimum depth of hardening layer	>1.2 mm
Distance between two indentation centers	4 mm
Distance between indentation center and sample edge	8 mm

**Table 2:** Indentation dimensionless parameters.

*h*_r_/*h*_m_	*P*_m_/(*A*_c_*E*)	*σ*_r_ *σ*_y_	*E*_e_/*E*_t_

**Table 3:** Factors and levels of orthogonal design.

	Young’s Modulus (*E*) GPa	Yield Strength (*σ*_y_) MPa	Work Hardening Exponent (*n*)	Density (*ρ*) g/cm^−3^	Poisson’s Ratio (*ν*)	Frictional Coefficient (*μ*)
1	40	50	0.0	1.5	0.24	0
2	130	225	0.1	6.0	0.28	0.1
3	220	400	0.2	10.5	0.32	0.2
4	310	575	0.3	15.0	0.36	0.3
5	400	750	0.4	19.5	0.40	0.4
Range	360	700	0.4	18.0	0.16	0.4

**Table 4:** Range analysis of simulation results of orthogonal design.

	$\bar{K_{\mathrm{ij}}}$	*P*_m_ (N)	*E*_k_ (mJ)	*h*_m_ (mm)	*h*_r_ (mm)	*a*_c_ (mm)
Young’s modulus (*E*) (GPa)	$\bar{K_{11}}$	1650.01	30.76	0.134	0.099	0.726
	$\bar{K_{12}}$	2123.49	17.29	0.102	0.088	0.662
	$\bar{K_{13}}$	2326.45	12.70	0.088	0.078	0.627
	$\bar{K_{14}}$	2445.35	10.52	0.080	0.074	0.609
	$\bar{K_{15}}$	2458.86	9.67	0.079	0.073	0.605
	*R*_1_	808.85	21.09	0.055	0.026	0.121
	*SE*_1_	150.255	3.875	0.0102	0.0049	0.0224
Yield strength (*σ*_y_) (MPa)	$\bar{K_{21}}$	1225.91	2.49	0.164	0.160	0.873
	$\bar{K_{22}}$	1967.00	10.66	0.097	0.086	0.666
	$\bar{K_{23}}$	2335.97	17.61	0.081	0.066	0.600
	$\bar{K_{24}}$	2599.04	22.71	0.073	0.054	0.560
	$\bar{K_{25}}$	2876.25	27.46	0.067	0.046	0.529
	*R*_2_	1650.34	24.97	0.097	0.114	0.344
	*SE*_2_	286.197	4.415	0.0176	0.0205	0.0613
work hardening exponent (*n*)	$\bar{K_{31}}$	1795.49	8.03	0.120	0.111	0.723
	$\bar{K_{32}}$	1987.09	10.80	0.106	0.095	0.682
	$\bar{K_{33}}$	2316.14	15.93	0.094	0.080	0.641
	$\bar{K_{34}}$	2327.00	20.27	0.085	0.068	0.606
	$\bar{K_{35}}$	2578.45	25.90	0.090	0.058	0.577
	*R*_3_	782.96	17.86	0.041	0.053	0.146
	*SE*_3_	138.166	3.214	0.0063	0.0094	0.0261
Density (*ρ*) g/cm^−3^	$\bar{K_{41}}$	2257.05	14.14	0.107	0.096	0.668
	$\bar{K_{42}}$	2316.61	15.17	0.089	0.076	0.628
	$\bar{K_{43}}$	2265.00	15.73	0.099	0.086	0.651
	$\bar{K_{44}}$	2184.22	17.14	0.089	0.072	0.626
	$\bar{K_{45}}$	1981.29	18.76	0.099	0.082	0.655
	*R*_4_	335.31	4.62	0.018	0.024	0.042
	*SE*_4_	58.804	0.805	0.0034	0.0042	0.0081
Poisson’s ratio (*ν*)	$\bar{K_{51}}$	2385.19	14.45	0.105	0.094	0.661
	$\bar{K_{52}}$	2221.59	15.63	0.097	0.084	0.652
	$\bar{K_{53}}$	2148.04	15.37	0.086	0.071	0.619
	$\bar{K_{54}}$	2163.43	18.04	0.104	0.088	0.664
	$\bar{K_{55}}$	2085.92	17.45	0.091	0.074	0.633
	*R*_5_	299.27	3.59	0.019	0.023	0.045
	*SE*_5_	50.897	0.672	0.0037	0.0043	0.0086
Frictional coefficient (*μ*)	$\bar{K_{61}}$	2344.26	13.03	0.107	0.097	0.667
	$\bar{K_{62}}$	2305.45	14.82	0.088	0.074	0.624
	$\bar{K_{63}}$	2114.05	17.74	0.094	0.078	0.643
	$\bar{K_{64}}$	2131.17	17.40	0.093	0.077	0.636
	$\bar{K_{65}}$	2109.24	17.94	0.102	0.086	0.658
	*R*_6_	235.02	4.90	0.019	0.023	0.043
	*SE*_6_	51.132	0.969	0.0034	0.0042	0.0077

**Table 5:** Influence of various factors on indentation parameters.

		*P*_m_ (N)	*E*_k_ (mJ)	*h*_m_ (mm)	*h*_r_ (mm)	*a*_c_ (mm)
*E* (GPa)	↑	↑	↓	↓	↓	↓
*σ*_y_ (MPa)	↑	↑	↑	↓	↓	↓
*n*	↑	↑	↑	↓	↓	↓

**Table 6:** Comparison between elastic modulus calculated by inversion and reference value.

Reference value (*E*) (GPa)	40	130	220	310	400
Inverse value (*E*) (GPa)	41.67	128.74	210.86	305.85	437.58
Relative error (%)	4.18	0.97	4.15	1.34	9.40

**Table 7:** Range analysis of simulation results of orthogonal design for 18 groups of virtual materials.

*R*′_j_		*P*_m_ (N)	*E*_k_ (mJ)	*h*_m_ (mm)	*h*_r_ (mm)	*a*_c_ (mm)
E	*R*′_1_	1076.03	18.23	0.030	0.057	0.131
*σ*_y_	*R*′_2_	1545.84	25.22	0.126	0.108	0.380
*n*	*R*′_3_	954.04	16.56	0.065	0.053	0.173
ρ	*R*′_4_	173.94	5.81	0.010	0.009	0.016
v	*R*′_5_	111.51	3.87	0.020	0.023	0.066
μ	*R*′_6_	256.89	2.89	0.027	0.024	0.057
*σ*_r/_ *σ*_y_	*R*′_7_	372.43	3.33	0.026	0.027	0.076

**Table 8:** Parameter fitting results of dimensionless function of *σ*_r_/*σ*_y_ to *E*_r_/*E*_o_.

Materials	*c*_0_	*c*_1_	*c*_2_	*c*_3_
304	0.79176	−0.03650	−0.00974	−0.00110
35Cr2Ni4MoA	0.94329	−0.14556	−0.04257	−0.00471
TC4-DT	1.04121	−0.12689	−0.08332	−0.04528

**Table 9:** Residual stress of measuring point of impact indentation method.

Measure Point	1	2	3	4	5	6	7
*σ*_r_ (MPa)	224.36	264.49	281.97	317.17	301.09	321.57	308.49

**Table 10:** Residual stress at the calibration measuring point of TC4-DT tested forging.

Number	*σ*_1_ (MPa)	*σ*_2_ (MPa)	*σ*_r_ (MPa)	*φ* (°)
①	219.72	196.05	208.89	−9.22
②	352.97	301.03	330.08	17.78

**Table 11:** Matching overall residual stress characteristic values of TC4-DT forgings in different states.

	TC4-DT Die Forging		TC4-DT Heat Treatment (Quenching)		TC4-Dtsecondary Annealing	
	Max (MPa)	Min (MPa)	Max (MPa)	Min (MPa)	Max (MPa)	Min (MPa)
Residual maximum principal stress	359.47	−101.16	415.14	−136.17	249.35	−125.15
Residual intermediate principal stress	316.64	−404.08	371.17	−493.70	217.99	−390.47
Residual minimum principal stress	72.89	−470.51	103.27	−539.23	50.14	−424.52
Residual equivalent stress	390.33	0.0241	432.50	0.0189	340.80	0.0119
Residual shear stress	49.306	−47.669	47.107	35.601	26.344	−23.593
Total deformation	9.670 mm	0.776 mm	9.979 mm	0.509 mm	5.958 mm	0.823 mm

## Data Availability

The original contributions presented in the study are included in the article, further inquiries can be directed to the corresponding author.
